# Safety, Tolerability, Pharmacokinetics, and Antimalarial Activity of the Novel *Plasmodium* Phosphatidylinositol 4-Kinase Inhibitor MMV390048 in Healthy Volunteers

**DOI:** 10.1128/AAC.01896-19

**Published:** 2020-03-24

**Authors:** Phumla Sinxadi, Cristina Donini, Hilary Johnstone, Grant Langdon, Lubbe Wiesner, Elizabeth Allen, Stephan Duparc, Stephan Chalon, James S. McCarthy, Ulrike Lorch, Kelly Chibale, Jörg Möhrle, Karen I. Barnes

**Affiliations:** a Division of Clinical Pharmacology, Department of Medicine, University of Cape Town, Cape Town, South Africa; b UCT MRC Collaborating Centre for Optimising Antimalarial Therapy, University of Cape Town, Cape Town, South Africa; c Medicines for Malaria Venture, Geneva, Switzerland; d HJ-Clinical Trial Consultancy, George, South Africa; e PTx Solutions Limited, London, United Kingdom; f QIMR Berghofer Medical Research Institute, Brisbane, Australia; g Richmond Pharmacology, London, United Kingdom; h Drug Discovery and Development Centre (H3D) and South African Medical Research Council Drug Discovery and Development Research Unit, University of Cape Town, Cape Town, South Africa

**Keywords:** malaria, *Plasmodium falciparum*, MMV390048, first-in-human, safety, pharmacokinetics, volunteer infection study, phosphatidylinositol-4-kinase

## Abstract

MMV390048 is a novel antimalarial compound that inhibits Plasmodium phosphatidylinositol-4-kinase. The safety, tolerability, pharmacokinetic profile, and antimalarial activity of MMV390048 were determined in healthy volunteers in three separate studies. A first-in-human, double-blind, randomized, placebo-controlled, single-ascending-dose study was performed. Additionally, a volunteer infection study investigated the antimalarial activity of MMV390048 using the Plasmodium falciparum induced blood-stage malaria (IBSM) model. Due to the high pharmacokinetic variability with the powder-in-bottle formulation used in both of these studies, a third study was undertaken to select a tablet formulation of MMV390048 to take forward into future studies. MMV390048 was generally well tolerated when administered as a single oral dose up to 120 mg, with rapid absorption and a long elimination half-life. Twelve adverse events were considered to be potentially related to MMV390048 in the first-in-human study but with no obvious correlation between these and MMV390048 dose or exposure. Although antimalarial activity was evident in the IBSM study, rapid recrudescence occurred in most subjects after treatment with 20 mg MMV390048, a dose expected to be subtherapeutic. Reformulation of MMV390048 into two tablet formulations (tartaric acid and Syloid) resulted in significantly reduced intersubject pharmacokinetic variability. Overall, the results of this study suggest that MMV390048 is well tolerated in humans, and the pharmacokinetic properties of the compound indicate that it has the potential to be used for antimalarial prophylaxis or inclusion in a single-dose cure. MMV390048 is currently being tested in a phase 2a study in Ethiopian adults with acute, uncomplicated falciparum or vivax malaria monoinfection. (The three clinical trials described here were each registered with ClinicalTrials.gov as follows: first-in-human study, registration no. NCT02230579; IBSM study, registration no. NCT02281344; and formulation optimization study, registration no. NCT02554799.)

## Abstract (precis)

MMV390048 is a novel antimalarial compound that inhibits Plasmodium phosphatidylinositol-4-kinase. The safety, tolerability, pharmacokinetic profile, and antimalarial activity of MMV390048 were determined in healthy volunteers in three separate studies. A first-in-human, double-blind, randomized, placebo-controlled, single-ascending-dose study was performed. Additionally, a volunteer infection study investigated the antimalarial activity of MMV390048 using the Plasmodium falciparum induced blood-stage malaria (IBSM) model.

## INTRODUCTION

A loss of efficacy of key artemisinin-based combination therapies in the Greater Mekong subregion highlights the urgent need for novel antimalarials (1). Resistance is now widely prevalent across at least six countries to both the fast-acting artemisinins and to their partner drugs (2). Drug resistance is a major threat to achieving malarial control and elimination goals as articulated by the World Health Organization (3). New antimalarials with novel mechanisms of action are urgently needed to combat parasite drug resistance and reduce malaria morbidity and mortality.

Several antimalarial candidates are currently in development (4, 5). One of these is MMV390048, an aminopyridine discovered through optimization of a high-throughput screening hit compound (6). MMV390048 selectively inhibits *Plasmodium* phosphatidylinositol-4-kinase (PI4K) (7), which is an attractive target for antimalarial drug development because it is required across all *Plasmodium* life cycle stages (8). Preclinical studies predicted a long elimination half-life of ∼90 h in humans, good oral bioavailability, and that a single oral dose of 80 to 100 mg would maintain the concentration of MMV390048 above therapeutic levels for 8 days, a duration estimated to result in cure (approximately 4 asexual Plasmodium falciparum life cycles) (9). Overall, results from preclinical studies indicated that MMV390048 has potential for inclusion in a single-dose antimalarial therapy (10, 11) and supported progression of the compound to clinical development.

In this report, we present results from three phase 1 clinical studies of MMV390048. The safety, tolerability, and pharmacokinetic profile of MMV390048 in a powder-in-bottle formulation were assessed in a first-in-human clinical trial. The antimalarial activity of the MMV390048 powder-in-bottle formulation was also tested in a volunteer infection study using the induced blood-stage malaria (IBSM) model, whereby healthy subjects are inoculated with blood-stage P. falciparum. Finally, the pharmacokinetic profiles of two MMV390048 tablet formulations were assessed in a formulation optimization study.

## RESULTS

### Study subjects.

**FIG 1:**
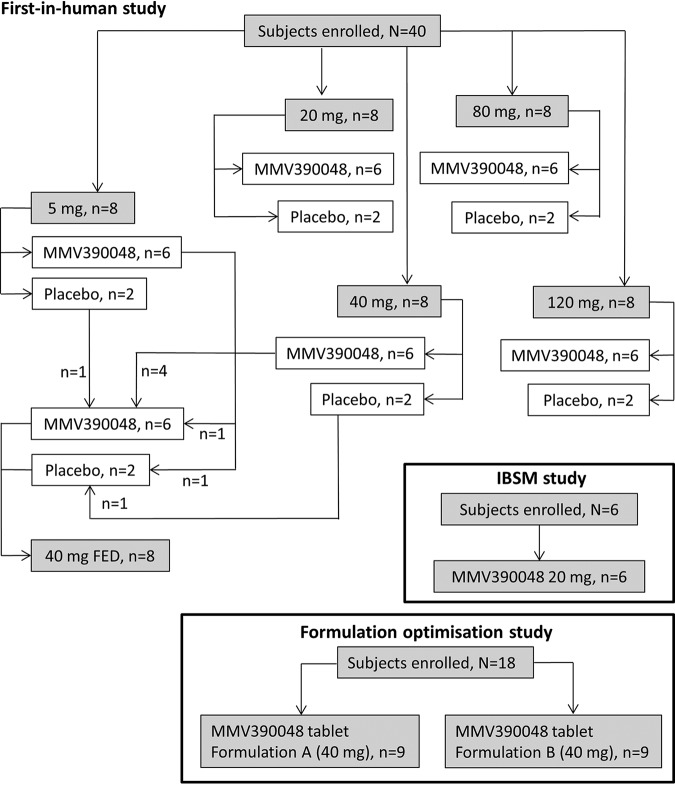
Subject flowchart for the first-in-human, induced blood-stage malaria (IBSM), and formulation optimization studies. Formulation A, tartaric acid tablets; formulation B, Syloid tablets.

The subject flow for the three studies is presented in Fig. 1, and subject demographics are summarized in Table 1. Forty subjects were enrolled in the first-in-human study in five sequential, fasted-dose cohorts (5 mg, 20 mg, 40 mg, 80 mg, or 120 mg). Within each cohort, subjects were dosed with either MMV390048 powder-in-bottle formulation (6 subjects) or placebo (2 subjects). A sixth-dose cohort (40 mg FED) was dosed under fed conditions and was composed of subjects who had previously received treatment as part of the 5-mg or 40-mg fasted-dose cohorts (Fig. 1). The majority of subjects were male (36/42 [86%]), and most self-declared their ethnic identity as black (31/42 [74%]) (Table 1). One subject (120-mg-dose cohort) used undisclosed prohibited prior medication (carbamazepine and an unknown traditional Chinese treatment) for an undisclosed neurological disease (see “Safety,” below, for more details). It was retrospectively confirmed that this subject took carbamazepine until admission. Pharmacokinetic data from this subject were therefore excluded from analyses.

**TABLE 1:** Demographic and baseline characteristics by study and treatment group

Patient characteristic	First-in-human study							IBSM^*a*^ study, 20 mg (*n* = 6)	Formulation optimization study, 40 mg^*b*^	
	5 mg (*n* = 6)	20 mg (*n* = 6)	40 mg (*n* = 6)	80 mg (*n* = 6)	120 mg (*n* = 6)	40 mg FED (*n* = 6)	Placebo (*n* = 12)		Form. A (*n* = 9)	Form. B (*n* = 9)
Sex (no. [%])										
Male	6 (100)	4 (67)	4 (67)	5 (83)	6 (100)	4 (67)	11 (92)	6 (100)	9 (100)	9 (100)
Female	0	2 (33)	2 (33)	1 (17)	0	2 (33)	1 (8)	0	0	0
Race (no. [%])										
Black	4 (67)	4 (67)	4 (67)	4 (67)	6 (100)	4 (67)	9 (75)	0	1 (11)	1 (11)
Mixed race	1 (17)	2 (33)	2 (33)	2 (33)	0	2 (33)	2 (17)	0	2 (22)	0
Caucasian	0	0	0	0	0	0	1 (8)	6 (100)	5 (56)	7 (78)
Other	1 (17)	0	0	0	0	0	0	0	1 (11)	1 (11)
Age (yr)										
Mean	29.3	34.0	37.0	30.0	23.7	34.0	32.7	25.3	32.6	31.0
SD	11.0	10.0	7.6	12.1	4.7	10.7	11.7	4.0	10.4	11.5
Ht (cm)										
Mean	171.8	167.3	171.3	168.7	169.8	170.7	170.5	182.3	182.4	175.8
SD	6.6	8.0	7.5	4.9	5.8	7.2	8.1	7.5	5.3	2.9
Wt (kg)										
Mean	69.1	69.8	72.4	67.5	66.4	73.6	65.8	81.4	85.9	71.5
SD	9.8	14.8	14.0	9.2	11.7	12.5	11.9	16.6	10.5	8.1
BMI^*c*^ (kg/m^2^)										
Mean	23.4	24.7	24.6	23.9	23.1	25.2	22.6	24.5	25.7	23.1
SD	3.2	3.2	4.1	4.4	4.6	3.3	3.0	4.8	2.2	2.1

a IBSM, induced blood-stage malaria study.

b Form. A, formulation A (tartaric acid tablets); Form. B, formulation B (Syloid tablets).

c BMI, body mass index.

A total of 6 subjects were enrolled in the IBSM study in a single-dose cohort (Fig. 1). The planned sample size of 8 subjects per dose cohort was not achieved due to recruitment limitations. All 6 subjects were inoculated with blood-stage P. falciparum and dosed with 20 mg MMV390048 powder-in-bottle formulation (Fig. 1). All subjects were male and Caucasian (Table 1).

Eighteen subjects were enrolled in the formulation optimization study and were dosed with 40 mg MMV390048 in either a tartaric acid (formulation A; *n* = 9) or Syloid (formulation B; *n* = 9) tablet formulation (Fig. 1). All subjects were male, and most were Caucasian (Table 1).

### Safety.

A summary of adverse events (AEs) recorded for each study is presented in Table 2, and a detailed list is included in Tables S4 to S6 in the supplemental material.

**TABLE 2:** Summary of adverse events by study and treatment group

Adverse event data by type and group^*a*^	First-in-human study							IBSM^*b*^ study, 20 mg (*n* = 6)	Formulation optimization study, 40 mg^*f*^	
	5 mg (*n* = 6)	20 mg (*n* = 6)	40 mg (*n* = 6)	80 mg (*n* = 6)	120 mg (*n* = 6)	40 mg FED (*n* = 6)	Placebo (*n* = 12)		Form. A (*n* = 9)	Form. B (*n* = 9)
No. (%) of subjects with adverse events										
All AEs	4 (67)	5 (83)	5 (83)	6 (100)	6 (100)	6 (100)	11 (92)	5 (83)	4 (44)	3 (33)
Treatment related	0	3 (50)	3 (50)	2 (33)	1 (17)	2 (33)	2 (17)	0	0	2 (22)
Grade 2 AEs	0	4 (67)	3 (50)	5 (83)	2 (33)	2 (33)	4 (33)	3 (50)	0	1 (11)
Treatment related	0	1 (17)	1 (17)	0	1 (17)	1 (17)	0	0	0	0
Grade 3 AEs	0	0	1 (17)^*c*^	0	0	1 (17)^*c*^	1 (8)^*d*^	0	0	0
SAEs	0	0	0	0	1 (17)^*e*^	0	0	0	0	0
No. of adverse events										
All AEs	11	12	19	20	11	22	32	32	4	7
Treatment related	0	3	3	3	1	2	3	0	0	2
Grade 2 AEs	0	5	4	6	3	2	5	9	0	1
Treatment related	0	1	1	0	1	1	0	0	0	0
Grade 3 AEs	0	0	1^*c*^	0	0	1^*c*^	1^*d*^	0	0	0
SAEs	0	0	0	0	1^*e*^	0	0	0	0	0

a AEs, adverse events; SAEs, serious adverse events. The Common Terminology Criteria for Adverse Events (CTCAE 4.03) was used to grade the severity of adverse events (grade 1 to 5). No adverse events of grade 4 or 5 severity were reported. All grade 3 adverse events and the serious adverse event were considered to be possibly related to the study treatment.

b IBSM, induced blood-stage malaria.

c The grade 3 AEs recorded in the 40-mg and 40-mg-FED cohort relate to the same subject. That is, a single subject experienced a grade 3 AE when enrolled in the 40-mg cohort and another grade 3 AE when enrolled in the 40-mg-FED cohort. The AE was neutropenia in both instances.

d The grade 3 AE recorded in the placebo cohort was bullous dermatitis.

e The SAE recorded in the 120-mg cohort was generalized myoclonus.

f Form. A, formulation A (tartaric acid tablets); Form. B, formulation B (Syloid tablets).

**(i) First-in-human study.** A total of 127 AEs were reported, which were predominantly mild in severity (99/127 [78%]). The incidence and severity of AEs were similar between treatment groups. Fifteen AEs reported in 13 subjects were considered to be related to the investigational product; three of these AEs occurred in two subjects who received placebo. There was no apparent relationship between the MMV390048 dose or exposure and the incidence or severity of AEs considered to be treatment related. No trends in inhibin B, follicle-stimulating hormone (FSH), luteinizing hormone (LH), or testosterone levels were identified over the course of the study across the different dose groups (Fig. S1).

The only serious AE (SAE) reported in this study was generalized myoclonus approximately 24 h after dosing with 120 mg MMV390048, which occurred in a subject with an undisclosed history of epilepsy. This episode resolved after treatment with valproate and clonazepam. An electroencephalogram at the time showed a pattern consistent with a primary generalized form of epilepsy, such as juvenile myoclonic epilepsy. Although the subject had denied any past medical history or any medication use repeatedly, a subsequent search of the subject’s medical records disclosed a 7-year history of poorly controlled epilepsy and generalized cerebral atrophy, with approximately monthly episodes of generalized myoclonus. This subject had commenced treatment with carbamazepine in November 2013 but had voluntarily interrupted the undisclosed treatment when enrolling in the study. The principal investigator and neurologist consulted assessed the SAE as a preexisting condition, with the generalized myoclonus probably related to sleep deprivation at the time, and possibly exacerbated by a subtherapeutic level of carbamazepine and, possibly, MMV390048.

Other AEs considered to be related to MMV390048 were PR prolongation detected on electrocardiogram (ECG), decrease in blood pressure (to 87/52 mm Hg), dizziness, photophobia, malaise, and dysgeusia that were all mild in severity, diarrhea (2 cases in 2 subjects) and papular urticaria that were both moderate in severity, and severe neutropenia. The severe neutropenia (2 events in one subject) occurred 1 day after dosing with 40 mg MMV390048 in the fasted and then fed state. This subject, self-identified as a black African, had a nonclinically significant decreased neutrophil count at screening (1.85 × 10^9^ liter^−1^) and before dosing on both occasions (1.65 × 10^9^ liter^−1^ and 1.90 × 10^9^ liter^−1^ for fasted and fed states, respectively), consistent with benign ethnic neutropenia (12). The lowest observed neutrophil counts were observed 24 h after dosing (fasted state, 0.77 × 10^9^ liter^−1^; fed state, 0.97 × 10^9^ liter^−1^). Neutrophil counts returned to a level of >1.5 × 10^9^ liter^−1^ 4 days (fasted) and 13 days (fed) after MMV390048 dosing.

The case of moderate papular urticaria (bilateral on upper arms) occurred 14 days after dosing with 20 mg MMV390048 and was not associated with eosinophilia or other clinically significant abnormalities. The episode lasted 20 days and responded to treatment with topical hydrocortisone. The case of mild PR prolongation (maximum 285 ms) occurred 24 h after dosing with 20 mg MMV390048 and was still ongoing at the end-of-study follow-up visit (day 79) despite negligible plasma concentrations of MMV390048 at this point. The subject had a PR interval of 200.3 ms upon admission to the unit the day prior to MMV390048 dosing. No QT prolongation (QTcF, >450 ms) was recorded for any subject during the study, and no other trends or concerns with respect to ECG results were observed.

**(ii) IBSM study.** A total of 32 AEs were reported in 5 of the 6 subjects which were predominantly mild in severity (23/32 [71.9%]). The most common AE was headache (7 cases in 4 subjects). Most AEs were deemed to be related to malaria (81.3%); none were considered to be related to MMV390048. There were no SAEs or severe AEs reported and no clinically significant abnormal laboratory safety parameters or ECG findings.

**(iii) Formulation optimization study.** A total of 11 AEs were reported in 7 of the 18 subjects. There was one case of upper respiratory infection that was of moderate intensity (grade 2); all other AEs were mild. No SAEs were reported. Two AEs were deemed related to MMV390048, both reported in subjects in the Syloid tablet cohort; one was a headache occurring ∼7 h after dosing, which resolved within 3 h without treatment, and the other was a symmetrical, raised, pruritic, cutaneous rash over the medial aspect of both knees, which occurred 3 days after dosing and resolved spontaneously within 1 h. No clinically significant changes from baseline were seen in biochemistry, hematology, coagulation, urinalysis, vital signs, or ECG parameters.

### Pharmacokinetics.

**FIG 2:**
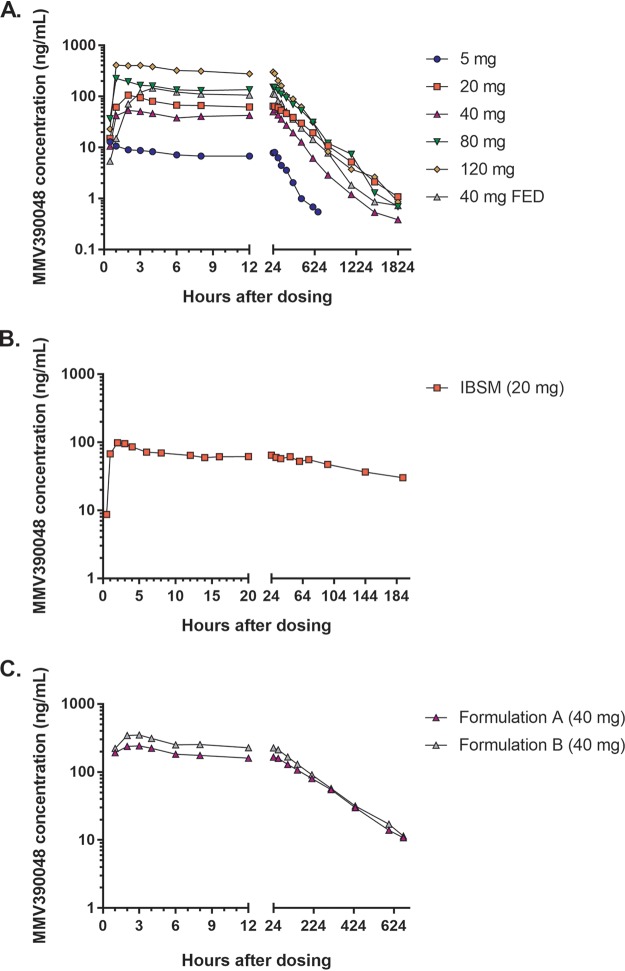
MMV390048 plasma concentration-time profiles by study and treatment group. (A to C) Plots represent the geometric mean of each treatment group in the first-in-human study (A), IBSM study (B), and formulation optimization study (C). IBSM, induced blood-stage malaria study; formulation A, tartaric acid tablets; formulation B, Syloid tablets.

In the first-in-human study, peak and total plasma exposures of MMV390048 generally appeared to increase with increasing doses (Fig. 2). However, considerable intersubject variability within each cohort was seen for all pharmacokinetic parameters (Table 3 and Fig. S2). The 20-mg cohort showed the least intersubject variability but a disproportionally high exposure with respect to other dose cohorts. The elimination half-life of MMV390048 (>149 h) was longer than was predicted from preclinical studies (90 h). The median time of maximum concentration (*T*_max_) was 1 to 2.5 h after dosing for the fasted cohorts but was longer for the 40-mg fed cohort (4 h). Furthermore, administration of an FDA-prescribed high-fat breakfast (13) prior to dosing reduced intersubject variability for all pharmacokinetic parameters in comparison to the equivalent dose administered fasted, although variability was still moderate. Investigations were performed to assess whether any manufacturer or site factors explained or contributed to the high levels of interindividual variability observed. It was concluded that neither dosing nor packaging or preparation of MMV390048 were likely to be the source of variability.

**TABLE 3:** MMV390048 pharmacokinetic parameters by study and treatment group^*a*^

Dose (mg) by study	*C*_max_ (ng ml^−1^)	AUC_0–inf_ (ng·h ml^−1^)	CL/F (liters h^−1^)	*V*_z_/F (liters)	*t*_1/2_ (h)	*T*_max_ (h)
First-in-human study						
5 (*n* = 6)	15.6 (109.4)	2,144.0 (76.3)	2.3 (76.3)	519.8 (63.5)	154.5 (51.0)	1.0 (0.5-48.1)
20 (*n* = 6)	136.2 (24.3)	33,274.0 (17.1)	0.6 (17.1)	265.5 (7.2)	306.1 (17.3)	2.0 (1.0-3.0)
40 (*n* = 6)	74.4 (128.1)	17,594.9 (86.8)	2.3 (86.8)	624.2 (63.0)	190.3 (64.7)	2.5 (0.5-48.0)
40 FED (*n* = 6)	154.7 (53.4)	34,494.7 (67.5)	1.2 (67.5)	373.4 (53.4)	223.2 (48.3)	4.0 (2.0-4.0)
80 (*n* = 6)	237.7 (110.3)	62,003.3 (46.0)	1.3 (46.0)	444.4 (64.0)	238.7 (45.0)	1.0 (1.0-96.0)
120 (*n* = 5)	517.8 (85.5)	82,644.5 (165.8)	1.5 (165.8)	431.8 (101.1)	206.1 (32.9)	1.0 (1.0-3.0)
IBSM study						
20 (*n* = 6)	105.8 (42.4)	15,928.8 (43.7)	NC	NC	NC	2.0 (1.0-3.0)
Formulation optimization study						
Form. A 40 (*n* = 9)	271.0 (22.3)	49,726.2 (53.5)	0.8 (53.5)	207.9 (20.7)	179.1 (37.6)	3.0 (1.0-4.0)
Form. B 40 (*n* = 9)	368.3 (18.0)	57,608.2 (48.6)	0.7 (48.6)	149.4 (29.3)	149.1 (44.0)	3.0 (2.0-3.0)

a Data are presented as the geometric means (coefficient of variation [%]), except for the *T*_max_, which is the median (range). IBSM, induced blood-stage malaria; Form. A, formulation A (tartaric acid tablets); Form. B, formulation B (Syloid tablets); *C*_max_, peak plasma concentration; AUC_0–inf_, area under the concentration-time curve from 0 h to infinity; CL/F, apparent clearance; *V*_z_/F, volume of distribution; *t*_1/2_, elimination half-life; *T*_max_, time at which *C*_max_ is reached; NC, not calculated (CL/F, *V*_z_/F, and *t*_1/2_ were not calculated for the IBSM study because the time interval over which MMV390048 was measured was not sufficient [less than two half-lives]).

High intersubject variability was also observed in the pharmacokinetic parameters in the IBSM study (Table 3 and Fig. S3). MMV390048 plasma concentrations were only assayed in samples taken up to 192 h after MMV390048 dosing (Fig. 2). The remainder of the samples were not analyzed due to the significant variability observed and the sponsor’s decision to suspend the study to reformulate the compound.

Absorption of MMV390048 after ingestion of the two tablet formulations was rapid following dosing, with a median *T*_max_ of 3 h for both formulations (Table 3 and Fig. S3). The maximum concentration of drug (*C*_max_) and area under the concentration-time curve from zero to infinity (AUC_0–inf_) were higher in the Syloid tablet (368.3 ng ml^−1^ and 57,608 ng·h ml^−1^, respectively) than in the tartaric acid tablet (271.0 ng ml^−1^ and 49,726 ng·h ml^−1^, respectively). For both formulations, interindividual variability was relatively low for *C*_max_ (coefficient of variation [CV], 18.0 to 22.3%), and moderate for AUC_0–inf_ (CV, 48.6 to 53.5%). The geometric mean half-life (*t*_1/2_) was longer for the tartaric acid tablet (179 h) than for the Syloid tablet (149 h), although variability was similar (CV, 37.6 to 44.0%).

### Antimalarial activity in the P. falciparum induced blood-stage malaria model.

**FIG 3:**
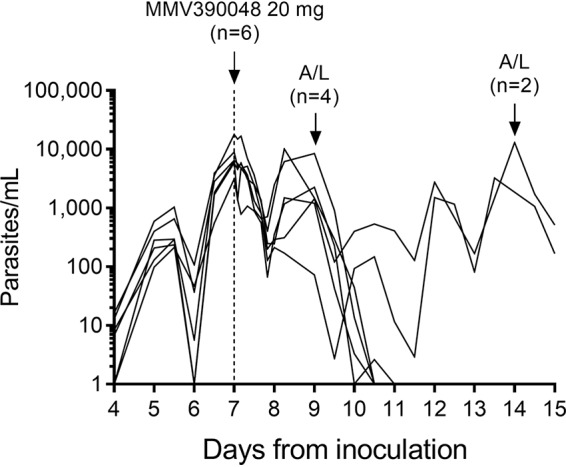
Time course of parasitemia in the induced blood-stage malaria (IBSM) study. Subjects (*n* = 6) were inoculated with ∼1,800 viable P. falciparum parasites on day 0, and a single dose of 20 mg MMV390048 was administered on day 7 (indicated by the vertical dashed line). Artemether-lumefantrine (A/L) was administered to 4 subjects on day 9 and to the remaining 2 subjects on day 14 in response to recrudescence of asexual parasitemia. Plots represent the parasitemia for each subject. For the purpose of graphing the parasitemia data on a logarithmic scale, time points at which parasites could not be detected were substituted with a value of 1 parasite/ml.

Parasitemia decreased initially in all subjects following administration of 20 mg MMV390048 (Fig. 3). However, recrudescence was observed in all subjects either 2 days (*n* = 4) or 7 days (*n* = 2) after MMV390048 dosing. The response to MMV390048 was prolonged in the two subjects who had the highest exposure; the area under the concentration-time curve from 0 h to the last time point measured (AUC_0–last_) was approximately 12,000 ng·h ml^−1^ for the two subjects who recrudesced 7 days after treatment, compared with approximately 6,000 to 9,000 ng·h ml^−1^ for the 4 subjects who recrudesced 2 days after treatment (data not shown). Subjects were treated per protocol with artemether-lumefantrine within 24 h of recrudescence. One subject showed evidence of gametocytemia at the time of artemether-lumefantrine treatment so was also treated with a single dose of 45 mg primaquine. All subjects were aparasitemic by the end of the study (day 28). Pharmacodynamic assessment of parasite clearance after MMV390048 treatment was not performed due to the smaller-than-planned sample size as a result of only one dose cohort being enrolled, rapid recrudescence, and the high intersubject pharmacokinetic variability associated with the powder-in-bottle formulation.

## DISCUSSION

In this report, we present the safety, pharmacokinetic profile, and antimalarial activity of MMV390048, the first *Plasmodium* PI4K inhibitor to reach clinical development. First-in-human studies are not typically conducted in low- and middle-income countries due to limitations in technical expertise and infrastructure (14). The successful completion of the MMV390048 first-in-human study at the University of Cape Town represents an important advance in this respect.

Overall, the results of the three clinical trials indicate that MMV390048 is generally well tolerated in healthy adult subjects up to a single oral dose of 120 mg. One SAE, generalized myoclonus, was reported in a subject with an undisclosed history of epilepsy. The subject’s cessation of his prescribed anticonvulsants and sleep deprivation were the most likely factors causing his seizure, although the potential of MMV390048 to lower the seizure threshold could not be excluded.

Severe neutropenia was observed in one subject on both occasions when dosed with 40 mg MMV390048 (once in a fasted state, once in a fed state). Although this subject was felt to have benign ethnic neutropenia (12), and no clinically significant abnormal neutrophil values were recorded in the IBSM study or formulation optimization study, neutrophil counts should be carefully monitored in future trials. All other AEs considered to be potentially related to MMV390048 were mild or moderate in severity, with no obvious correlation between the incidence or severity of these AEs and the dose or exposure of MMV390048. Additionally, other than diarrhea, which was reported for 2 subjects, all other AEs related to MMV390048 were observed in a single subject only. Collectively, these data support the further clinical development of MMV390048 for antimalarial prophylaxis or treatment.

The pharmacokinetic profiles of MMV390048 varied considerably between subjects within the same dose cohort in both the first-in-human and IBSM studies, where the powder-in-bottle formulation was used. Peak and total exposures generally increased with increasing doses (except the 20-mg cohort in the first-in-human study), but dose exposure was difficult to interpret, and linearity could not be established. Estimates of elimination half-life were highly variable within each cohort, resulting in significant uncertainty regarding the actual half-life of MMV390048. The variability was considered to be likely due to differential absorption of the powder-in-bottle formulation of MMV390048 between individual subjects rather than dosing, packaging, or preparation of the compound at either site.

The IBSM study started after the 5-mg and 20-mg dose cohorts of the first-in-human study were completed. Although preclinical work indicated that MMV390048 is active against all *Plasmodium* life cycle stages (9) and thus has the potential to target liver-stage infection, the current study focused on determining its activity against blood-stage parasites, as these cause the clinical features of malaria. An attractive characteristic of the IBSM model is that it can be conducted in parallel with an ongoing first-in-human study, allowing accrual of key pharmacokinetic and pharmacodynamic data in a short time frame. This enables rapid prediction of the potential clinically efficacious dose and hastens the process of drug development. Although there was evidence of antimalarial activity in the IBSM study, and notably that recrudescence was delayed in the two subjects with higher MMV390048 exposure, the high variability in exposure and response prevented progression to the evaluation of the 80- to 100-mg dose expected to be effective and the execution of the planned pharmacokinetic and pharmacodynamic modeling. Instead, it was decided to reformulate the compound.

A powder-in-bottle formulation was chosen for the first-in-human and IBSM studies since it represents a cost- and time-effective strategy to progress a compound from preclinical to clinical studies. Despite the pharmacokinetic variability observed with the powder-in-bottle formulation of MMV390048, the studies using this formulation led to confirmation that the compound was well tolerated and had antimalarial activity in humans and thus reduced the risk associated with investing in more sophisticated formulation work. Two tablet formulations (tartaric acid and Syloid) were selected from various tablet formulation prototypes based on their stability and dissolution performance in biorelevant media. Both tablet formulations exhibited substantially reduced variability in exposure and other pharmacokinetic parameter estimates, as well as a long elimination half-life that was greater than predicted in preclinical studies (90 h). The tartaric acid formulation was chosen to be progressed in further clinical trials due to its favorable pharmacokinetic profile and less-challenging manufacturing process compared to that with the Syloid formulation. The results from the fed cohort in the first-in-human study suggested that food intake may delay the absorption of MMV390048, but this needs to be confirmed by repeating a food effect study with the final clinical formulation.

Despite the significant pharmacokinetic variability observed with the powder-in-bottle formulation, the results reported here support the potential for MMV390048 to be included in a single-dose cure combination regimen (or chemoprophylaxis) for falciparum malaria. The preclinical efficacy study conducted in humanized severe combined immunodeficient mice infected with P. falciparum predicted an effective concentration to achieve 90% of the maximal kill rate (EC_90_) of 160 ng ml^−1^ (9). The long elimination half-life for MMV390048 in humans was confirmed in the clinical studies, and the geometric mean MMV390048 plasma concentration in the 120-mg-dose cohort (first-in-human study) remained above the EC_90_ for 6 days after dosing, despite the high variability of exposure with the powder-in-bottle formulation. Thus, parasiticidal concentrations of MMV390048 can be maintained for an extended period after administration of a single dose, potentially preventing recrudescence without the need for a multiple-dose treatment regimen. A follow-up IBSM study to characterize the pharmacodynamic antimalarial activity of the MMV390048 tablet formulation and to estimate the efficacious dose in humans has recently been completed, and the results will be published separately.

In summary, MMV390048 was well tolerated when administered as a single oral dose up to 120 mg in healthy volunteers and displayed reduced pharmacokinetic variability after reformulation of the powder-in-bottle to a tablet formulation. This antimalarial candidate has the potential to be used for prophylaxis or inclusion in a single-dose cure. MMV390048 is currently being tested in a phase 2a study in Ethiopian adults with acute, uncomplicated falciparum or vivax malaria monoinfection (ClinicalTrials.gov identifier NCT02880241).

## MATERIALS AND METHODS

### Clinical study design.

The first-in-human study was conducted at the University of Cape Town Clinical Research Centre (Cape Town, South Africa) between 30 April 2014 and 2 February 2015. This was a phase 1, adaptive, single-center, double-blind, randomized, placebo-controlled, ascending-dose study. The study was planned in two parts, with a single ascending dose and then a multiple ascending dose. However, the multiple-ascending-dose part was not executed due to the high variability in pharmacokinetic profiles observed during the single-ascending-dose part (see Results).

The second study was conducted at Q-Pharm (Brisbane, Australia) from 27 October 2014 to 19 December 2014. This was an IBSM, phase 1b, single-center, open-label study, in which a single dose of MMV390048 was administered to subjects previously inoculated with blood-stage P. falciparum. This study was planned to consist of two dose cohorts; however, a second cohort was not enrolled due to the high variability in pharmacokinetic profiles observed in the first cohort and in the first-in-human study (see Results). The IBSM study commenced when the first two dose cohorts of the first-in-human study had been completed. MMV390048 was administered in the first-in-human and IBSM studies in a powder-in-bottle formulation.

The third study was a formulation optimization study to test two MMV390048 tablet formulations (tartaric acid and Syloid). The formulation optimization study was conducted at Richmond Pharmacology Ltd. (London, United Kingdom) from 17 September to 28 October 2015. This was a phase 1, open-label, single-dose, parallel-group, two-cohort design study.

Healthy subjects aged 18 to 55 years were eligible for the studies (for the first-in-human study and formulation optimization study, men and women of nonchildbearing potential; for the IBSM study, malaria-naive men only). A complete list of the inclusion and exclusion criteria for each study is included in the supplemental material.

The first-in-human study was approved by the University of Cape Town, Faculty of Health Sciences Human Research Ethics Committee (approval number 009/2014). The IBSM study was approved by the QIMR Berghofer Medical Research Institute Human Research Ethics Committee (approval number H0509-046T [P922]). The formulation optimization study was approved by the National Research Ethics Service Committee London-Brent (approval number 15/LO/1415). The studies were registered with ClinicalTrials.gov (first-in-human study, NCT02230579; IBSM study, NCT02281344; formulation optimization study, NCT02554799). All studies were conducted according to the Declaration of Helsinki and the ICH Guideline for Good Clinical Practice. All subjects gave written, informed consent before screening.

### Randomization and blinding.

Subjects in the first-in-human study were randomized to either MMV390048 or placebo in a 3:1 ratio. Treatment identity was concealed by identical packaging and the appearance, odor, and taste of both MMV390048 and the placebo. Subjects in the two parallel cohorts of the formulation optimization study were randomized in a 1:1 ratio to one of the two MMV390048 tablet formulations. The randomization schedules were generated electronically by independent, unblinded statisticians using the PROC PLAN procedure in SAS. No randomization was performed in the IBSM study.

### Procedures.

**(i) First-in-human study.** The study was conducted in five fasted cohorts (5, 20, 40, 80, and 120 mg) and one fed 40-mg cohort. The initial dose of 5 mg was chosen in agreement with guidelines on dose selection for first-in-human clinical studies (15, 16) and was based on the no-observed-adverse-event level for the most sensitive species observed in preclinical toxicology studies. Doses administered to subsequent cohorts were determined *a priori* after consideration of interim human pharmacokinetic and safety data.

MMV390048 and placebo were each supplied as a powder for suspension packaged in white opaque high-density polyethylene bottles, with a volume capacity of 100 ml when reconstituted with deionized water (Almac Pharma Services, United Kingdom). The powder contained the following excipients: Avicel CL611, disodium edetate, sodium citrate dihydrate, methyl paraben, sucralose, povidone, and orange flavor. The reconstituted formulation consisted of the active ingredient MMV390048 in an aqueous suspension of 5 mg ml^−1^.

All subjects in the fasted cohorts were dosed on day 1 after a fasting period of ≥8 h. Water was prohibited from 2 h predose and then allowed *ad libitum* from 2 h postdose, and standardized meals were provided from 4 h postdose. In the first two cohorts (5 and 20 mg), a sentinel subcohort (*n* = 2) was dosed on day 1. The safety review team reviewed safety and pharmacokinetic data of these subjects up to day 3 before dosing the rest of these two cohorts and up to day 19 for all cohorts before dose escalation. Subjects in the 5-mg cohort were followed until day 29, while subjects in subsequent cohorts were followed until day 77, as the elimination half-life of MMV390048 in cohort 1 was found to be longer than predicted from preclinical data. Selected subjects from the fasted cohorts then received 40 mg in a fed state to evaluate the effect of food on the tolerability and pharmacokinetic profile of MMV390048. Subjects in the fed cohort had a high-fat breakfast 30 min before dosing, which occurred at least five half-lives after the fasted dose to ensure minimal carryover effect. The predose breakfast was in line with FDA guidelines (13) and was composed of approximately 800 to 1,000 calories (cal), with 150, 250, and 500 to 600 cal from protein, carbohydrate, and fat, respectively.

**(ii) IBSM study.** The dose of MMV390048 chosen (20 mg) was based on the pharmacokinetic, safety, and tolerability data available from the first-in-human study at the time the IBSM study commenced (when 5- and 20-mg-dose cohorts of the first-in-human study had been completed). This dose was below the estimated single dose required to achieve complete cure calculated from preclinical studies (80 to 100 mg) since recrudescence allows for characterization of the pharmacokinetic/pharmacodynamic relationship between MMV390048 and P. falciparum parasitemia. Subjects were inoculated intravenously on day 0 with erythrocytes infected with ∼1,800 viable chloroquine-sensitive P. falciparum 3D7 parasites (17). Fasted (≥8 h) subjects were dosed with MMV390048 when they reached the parasitemia threshold for treatment (∼1,000 parasites ml^−1^) and were followed up until the end-of-study visit on day 28.

**(iii) Formulation optimization study.** Subjects received one of two tablet formulations (formulation A, tartaric acid tablets; formulation B, Syloid tablets; manufactured by Quotient Clinical, United Kingdom) on day 1, after an ≥8-h fasting period, at a dose of 40 mg with 240 ml of water. Subjects were followed up until day 29.

### Safety.

Safety assessments were performed at protocol-specified times (the full schedule of events for each study is presented in Tables S1 to S3). Safety endpoints were the incidence, severity, and relationship to the investigational product (and inoculum for the IBSM study) of observed and self-reported adverse events (AEs), and changes from baseline in physical examination, vital signs, standard electrocardiograms (ECGs), continuous ECGs (first-in-human study only), and laboratory evaluation (hematology, coagulation parameters, hemolysis panel, clinical chemistry, and urinalysis) findings. Additionally, inhibin B, follicle-stimulating hormone (FSH), luteinizing hormone (LH), and testosterone were measured for endocrine assessment of testicular function in the first-in-human study since testicular toxicity was observed in preclinical toxicology studies conducted in rats. AE severity was evaluated according to the Common Terminology Criteria for Adverse Events version 4.03 (18) and the World Health Organization recommendations for grading acute and subacute toxic effects (19).

### Pharmacokinetics.

Blood samples were collected at the following time intervals after dosing to determine the MMV390048 plasma concentration and calculate pharmacokinetic parameters: for the first-in-human study, 0.5, 1, 2, 3, 4, 6, 8, 9, 12, 24, 48, 96, 144, 216, 312, 432, 600, and 672 h for the 5-mg cohort and additional 816 (day 35), 1,152 (day 49), 1,488 (day 63), and 1,824 (day 77) hours for the subsequent cohorts; for the IBSM study, 0.5, 1, 2, 3, 4, 6, 8, 12, 14, 16, 20, 24, 30, 48, 72, 96, 144, 192, 240, 336, 432, and 505 h; and for the formulation optimization study, 1, 2, 3, 4, 6, 8, 12, 24, 48, 96, 144, 216, 312, 432, 600, and 672 h.

Blood samples from the three studies were analyzed at the University of Cape Town Division of Clinical Pharmacology Laboratory. MMV390048 was extracted from plasma using a protein precipitation procedure and analyzed using a liquid chromatography-tandem mass spectrometry assay. The precision (total assay coefficients of variation) for MMV390048 during sample analysis was less than 8% at all quality control levels, including the limit of quantification, which was 0.5 ng ml^−1^. The full details of the assay are included in the supplemental material.

Noncompartmental pharmacokinetic analysis was performed using Phoenix WinNonlin Pro version 6.3 (Certara L.P., St. Louis, MO, USA) for the first-in-human and the formulation optimization studies, and using R version 3.2.0 for the IBSM study. Pharmacokinetic endpoints were the peak plasma concentration (*C*_max_), time at which *C*_max_ is reached (*T*_max_), area under the concentration-time curve from 0 h to infinity (AUC_0–inf_), elimination half-life (*t*_½_), apparent clearance (CL/F), and apparent volume of distribution (*V*_z_/F).

### Parasitemia measurement.

Parasitemia was monitored by collecting blood samples and performing quantitative PCR (qPCR) targeting the gene encoding P. falciparum 18S rRNA (20). Blood samples were collected daily from day 4 until parasites were detectable; twice daily until MMV390048 dosing; before MMV390048 dosing and 2, 4, 8, 12, 16, 20, 24, 30, 36, 48, 60, and 72 h after MMV390048 dosing; twice daily until parasitemia was not detected over a 48-h period; 3 times per week until artemether-lumefantrine treatment; and then at the end of the study (day 28).

### Sample size.

The planned sample size of the first-in-human study (8 subjects per cohort) was calculated based on the assumption that if a dose of the study treatment is associated with a risk of ≥50% for a toxicity-related event, the probability that this event will be observed in at least one of the six subjects receiving MMV390048 is >98%. The planned sample size of the IBSM study (8 subjects per cohort) was calculated as previously described (21). Due to the exploratory nature of the formulation optimization study, the planned sample size (9 subjects per cohort) was not based on formal statistical considerations but was deemed adequate to achieve the study objectives.

### Data availability.

The data that support the findings of this study are available from Medicines for Malaria Venture upon reasonable request.

## Supplementary Material

Supplemental file 1
