# Supplementary material for: Safety, Tolerability, Pharmacokinetics, and Antimalarial Activity of the Novel Plasmodium Phosphatidylinositol 4-Kinase Inhibitor MMV390048 in Healthy Volunteers
Source: Antimicrob Agents Chemother. 2020 Mar 24;64(4):e01896-19. doi: 10.1128/AAC.01896-19 (PMC7179259; doi:10.1128/AAC.01896-19)
Supplement: Supplemental file 1 [file AAC.01896-19-s0001.pdf]

Supporting information

Safety, tolerability, pharmacokinetics and antimalarial activity of the novel *Plasmodium* phosphatidylinositol 4-kinase inhibitor MMV390048 in healthy volunteers

Phumla Sinxadi, Cristina Donini, Hilary Johnstone, Grant Langdon, Lubbe Wiesner, Elizabeth Allen, Stephan Duparc, Stephan Chalon, James S. McCarthy, Ulrike Lorch, Kelly Chibale, Jörg Möhrle, Karen I. Barnes

Contents

Inclusion and exclusion criteria for subjects in each clinical trial 2

Inclusion/exclusion criteria for the first-in-human study 3

Inclusion/exclusion criteria for the IBSM study 8

Inclusion/exclusion criteria for the formulation optimisation study 13

Schedule of events for each clinical trial 17

Table S1: Schedule of events for the first-in-human study 18

Table S2: Schedule of events for the IBSM study 21

Table S3: Schedule of events for the formulation optimisation study 24

Assay for measuring MMV390048 concentrations in human plasma 27

Adverse events by MedDRA system organ class and preferred term for each clinical trial 29

Table S4: Adverse events for the first-in-human study 30

Table S5: Adverse events for the IBSM study 36

Table S6: Adverse events for the formulation optimisation study 38

Endocrinology results 40

Figure S1: Median concentration of follicle stimulating hormone, luteinizing hormone, testosterone and inhibin B over time by treatment group for male subjects 41

Individual subject MMV390048 plasma concentration results 43

Figure S2: Individual subject MMV390048 plasma concentration-time profiles for the first-in-human study 44

Figure S3: Individual subject MMV390048 plasma concentration-time profiles for the IBSM and formulation optimisation studies 46

Inclusion and exclusion criteria for subjects in each clinical trial

Inclusion/exclusion criteria for the first-in-human study

Inclusion criteria

Subjects eligible for inclusion in this trial had to fulfil all of the following criteria:

1. Completion of the written informed consent process.

2. Male and female (of non-childbearing potential) subjects age 18 to 55 years, in good health as determined by past medical history, physical examination, vital signs, electrocardiogram, and laboratory tests at screening.

[Women were considered post-menopausal and/or not of child-bearing potential if they had had at least 12 months of natural (spontaneous) amenorrhea with an appropriate clinical profile (e.g. age appropriate, history of vasomotor symptoms) or six months of spontaneous amenorrhea with local laboratory serum FSH levels commensurate for post-menopause; or had had surgical bilateral oophorectomy or bilateral salpingectomy (with or without hysterectomy) at least six months prior to screening. Women on contraceptives were excluded from the trial].

3. Haematology, clinical chemistry and urinalysis results at screening within the local laboratory reference range or, if outside the range, judged as not clinically significant by the investigator and confirmed and agreed upon by the medical monitor. Specific laboratory parameters that had to lie within the normal reference ranges (AST, ALT, LDH, total bilirubin, haptoglobin and haemoglobin) are detailed in Section 7.2 as exclusion criteria.

4. Body weight of at least 50 kg and a body mass index (BMI) within the range of 18-32 kg/m2.

5. Good peripheral venous access.

6. An ability to communicate well with the investigator, to understand and comply with the requirements of the trial.

7. Agreement to stay in contact with the trial site for the duration of the trial, provide updated contact information as necessary, and no plans for moving away from the trial area for the duration of the trial.

Exclusion criteria

Subjects that met any of the following criteria were not eligible for inclusion in this trial:

1. Any acute illness upon admission to the unit on Day -1 or prior to dosing on Day 1.

2. Use of any other investigational drug within 30 days or five half-lives (whichever is longer) prior to the first dose of MMV390048.

3. A history of hypersensitivity to any drugs.

4. A history of anaphylaxis or severe allergic reaction.

5. Resting vital signs (measured after 5 minutes in the supine position) at either screening or baseline outside of the following ranges:

• oral body temperature between 35.0 and 37.5 °C

• systolic blood pressure from 90 to 140 mmHg

• diastolic blood pressure from 50 to 90 mmHg

• pulse rate from 40 to 90 bpm.

6. Orthostatic changes in blood pressure and heart rate measurements greater than:

• 20 mmHg drop in systolic blood pressure

• 10 mmHg drop in diastolic blood pressure

• 20 bpm increase in heart rate

[Orthostatic changes were assessed by comparing supine measurements (after at least 5 minutes in the supine position) to measurements taken 3 minutes after standing up from the supine position.]

7. A history of clinically significant ECG abnormalities, or any of the following ECG abnormalities at either screening or baseline:

• PR >210 ms

• QRS complex >120 ms

• QTcF >450 ms or shortened QTcF less than 340 ms or a family history of long QT syndrome or sudden death

• Second or third degree atrioventricular block

• Incomplete, complete or intermittent bundle branch block

• Abnormal T wave morphology

• Left ventricular hypertrophy with repolarisation abnormalities

• Right ventricular hypertrophy.

8. History of malignancy of any organ system (other than localised basal cell carcinoma of the skin), treated or untreated, within the past five years, regardless of whether there was evidence of local recurrence or metastases.

9. Pregnant or nursing (lactating) women.

10. Women of child-bearing potential, defined as all women physiologically capable of becoming pregnant, including women whose career, lifestyle, or sexual orientation precluded

intercourse with a male partner and women whose partners had been sterilized by vasectomy or other means.

11. Fertile males, defined as all males physiologically capable of conceiving offspring unless the subject agreed to the use of condoms and ensuring that his partner(s) was either not of child-bearing potential or used a highly effective method of contraception for the entire duration of the trial and for twelve weeks following the last IP administration.

[Oral contraceptives, injectable contraceptives or intra-uterine devices were acceptable as highly effective methods of contraception; women of child-bearing potential were defined in exclusion criterion no. 10.]

12. Smokers (use of tobacco products in the previous three months). Serum cotinine levels will be measured during screening and at baseline for all volunteers. A smoker is defined as a person who reports tobacco use and/or who has a serum cotinine level ≥25 ng/ml.

13. Use of any prescription drugs, herbal supplements, over-the-counter (OTC) medication or dietary supplements (vitamins included) within four weeks prior to initial dosing.

[If necessary (i.e. for an incidental and short-term need) paracetamol was acceptable at a dose of up to 4 g/day.]

14. Intake of grapefruit, grapefruit juice or other products containing grapefruit within 28 days of the first drug administration of the IP.

15. Excessive intake of caffeine drinks or energy drinks within 48 hours before admission defined as more than three 250 ml cups of coffee a day, equivalent to roughly 250 mg of caffeine.

16. Donation or loss of 400 ml or more of blood within eight weeks prior to screening or initial dosing.

17. Plasma donation (>100 ml) within 60 days prior to first dosing.

18. Haemoglobin levels below 12.5 g/dl (males) or 11.5 g/dl (females) at screening as determined by a full blood count.

19. Haptoglobin levels outside the reference range for the local trial laboratory.

20. Positive direct antiglobulin test (direct Coomb’s test).

21. ALT and/or AST and/or lactase dehydrogenase (LDH) and/or total bilirubin levels greater than the upper limit of normal (ULN) for the local trial laboratory.

22. Liver enzymes other than ALT, AST and LDH elevated ≥1.5 x ULN within two weeks prior to initial dosing.

23. Recent (within the last three years) and/or recurrent history of autonomic dysfunction (e.g. recurrent episodes of fainting, palpitations, etc.).

24. Recent (within three years of screening) and/or recurrent history of acute or chronic bronchospasm (including asthma and chronic obstructive pulmonary disease, treated or not treated).

25. A history of immunodeficiency diseases, including a confirmed positive HIV test result.

26. A positive Hepatitis B surface antigen (HBsAg) or Hepatitis C antibody test result.

27. A history of recurrent infections.

28. A history of endocrine disease, in particular adrenal disorders such as Cushing's syndrome or Addison's disease, or diabetes mellitus.

29. A history of Gilbert’s Syndrome.

30. A history of photosensitivity.

31. A history of any food allergies.

32. Any surgical or medical condition with the potential to significantly alter the absorption, distribution, metabolism, or excretion of drugs, or which might have jeopardised the safety of the subject or the objectives of the trial. The investigator made this determination in consideration of the subject’s medical history and/or clinical or laboratory evidence. Possible relevant conditions included the following:

• Inflammatory bowel disease, ulcers, gastrointestinal or rectal bleeding in the last 6 months;

• Major gastrointestinal tract surgery such as gastrectomy, gastroenterostomy, or bowel resection; or

• Pancreatic injury or pancreatitis in the last 6 months.

33. A history or presence of impaired renal function as indicated by clinically significantly abnormal creatinine or urea values, or abnormal urinary constituents (e.g. albuminuria).

34. History of urinary obstruction or difficulty in voiding at screening.

35. A history of drug or alcohol abuse within the 12 months prior to dosing, or evidence of such abuse as indicated by the tests and laboratory assays conducted during screening and/or baseline. (Unacceptable alcohol use was defined as the consumption of more than 21 units of alcohol per week (one unit being equivalent to 8-10g of ethanol, 285ml of beer or lager, one glass [125mL] of wine, or 25mL of spirits), or use resulting in a positive alcohol breath test at screening.)

36. Any clinically significant mental disorder that could limit the validity of informed consent or the subject’s ability to comply with protocol requirements.

Inclusion/exclusion criteria for the IBSM study

<u>Inclusion criteria</u>

Participants eligible for inclusion in this study were to fulfil all of the following inclusion criteria:

Demography

1. Male participants, between 18 and 55 years of age, inclusive, who did not live alone (from Day 0 until at least the end of the antimalarial drug treatment) and were contactable and available for the duration of the trial (maximum of 4 months).
2. Body weight, minimum 50.0 kg, body mass index (BMI) between 18.0 and 32.0 kg/m2, inclusive.

Health status

3. Certified as healthy by a comprehensive clinical assessment (detailed medical history and complete physical examination).
4. Normal vital signs after 10 minutes resting in supine position:
95 mmHg < systolic blood pressure (SBP) <140 mmHg,
50 mmHg < diastolic blood pressure (DBP) <90 mmHg,
40 bpm< heart rate (HR) <100 bpm.
5. Normal standard 12-lead ECG after 10 minutes resting in supine position; QTcF ≤450 ms with absence of second or third degree atrioventricular block or abnormal T wave morphology.
6. Laboratory parameters within the normal range, unless the Investigator considered an abnormality to be clinically irrelevant for healthy participants enrolled in this clinical investigation (for example, participants with asymptomatic mild hypercholesterolemia or glucose intolerance could be included). Serum creatinine, alkaline phosphatase, hepatic enzymes (aspartate aminotransferase, alanine aminotransferase), and total bilirubin (unless the participant had documented Gilbert syndrome) should not exceed the upper laboratory norm and haemoglobin had to be higher than the lower limit of the normal range.
7. Participants have to agree to use a double barrier method of contraception including condom plus diaphragm or condom plus IUD or condom plus stable oral/transdermal/injectable hormonal contraceptive by female partner for at least 14 days prior to the time of the first dose of study drug through 90 days after the last dose of study drug. Abstinent participants have to agree starting a double barrier method if they start sexual relationships during the study and up to 90 days after the last dose of study drug.

Regulations

8. Had given written informed consent prior to undertaking any study-related procedure.

<u>Exclusion criteria</u>

Participants who met any of the following criteria were not eligible for inclusion in this study:

Medical history and clinical status

1. Any history of malaria or participation to a previous malaria challenge study.
2. Must not have travelled to or lived (>2 weeks) in a malaria-endemic country during the past 12 months or planned travel to a malaria-endemic country during the course of the study.
3. Had evidence of increased cardiovascular disease risk (defined as >10%, 5 year risk) as determined by the method of Gaziano et al. (5). Risk factors included sex, age, systolic blood pressure (mm/Hg), smoking status, BMI (kg/m2) and reported diabetes status.
4. History of splenectomy.

5. Presence or history of drug hypersensitivity, or allergic disease diagnosed (including seasonal rhinitis) and treated by a physician or history of a severe allergic reaction, anaphylaxis or convulsions following any vaccination or infusion.
6. Presence of current or suspected serious chronic diseases such as cardiac or autoimmune disease (HIV or other immunodeficiencies), insulin-dependent and NIDDM diabetes (excluding glucose intolerance if exclusion criteria 3 is met), progressive neurological disease, severe malnutrition, acute or progressive hepatic disease, acute or progressive renal disease, psoriasis, rheumatoid arthritis, asthma, epilepsy or obsessive compulsive disorder, skin carcinoma excluding non-spreadable skin cancers such as basal cell and squamous cell carcinoma, schizophrenia, bi-polar disease, or other severe (disabling) chronic psychiatric diagnosis.
7. History of photosensitivity.
8. Participants with history of schizophrenia, bi-polar disease, or other severe (disabling) chronic psychiatric diagnosis, including depression or receiving psychiatric drugs or who had been hospitalized within the past 5 years prior to enrolment for psychiatric illness, history of suicide attempt or confinement for danger to self or others.
9. Frequent headache and/or migraine, recurrent nausea, and/or vomiting (more than twice a month).
10. Presence of acute infectious disease or fever (e.g., sub-lingual temperature ≥38.5°C) within the 5 days prior to inoculation with malaria parasites.
11. Evidence of acute illness within the 4 weeks before trial prior to screening that the investigator deems may compromise participant’s safety.
12. Significant intercurrent disease of any type, in particular liver, renal, cardiac, pulmonary, neurologic, rheumatologic, or autoimmune disease by history, physical examination, and/or laboratory studies including urinalysis.
13. Participant had a clinically significant disease or any condition or disease that might affect drug absorption, distribution or excretion, e.g. gastrectomy, diarrhoea.
14. Participation in any investigational product study within the 12 weeks preceding the study.
15. Participation in any research study involving to be desired blood sampling, or blood donation to Red Cross (or other) blood bank during the 8 weeks preceding the reference drug dose in the study.
16. Participant unwilling to defer blood donations to the ARCBS for 6 months.
17. Blood donation, any volume, within one month before inclusion.
18. Medical requirement for intravenous immunoglobulin or blood transfusions.
19. Participant who had ever received a blood transfusion.
20. Symptomatic postural hypotension, irrespective of the decrease in blood pressure, or asymptomatic postural hypotension defined as a decrease in systolic blood pressure ≥20 mmHg within 2-3 minutes when changing from supine to standing position.
21. History or presence of alcohol abuse (alcohol consumption more than 40 g per day) or drug habituation, or any prior intravenous usage of an illicit substance.
22. Smoking more than 5 cigarettes or equivalent per day and unable to stop smoking during the confinement period in the study.
23. Ingestion of any poppy seeds within the 24 hours prior to the screening blood test (participants will be advised by phone not to consume any poppy seeds in this time period).
24. Excessive consumption of beverages containing xanthine bases, including red bull, chocolate, etc. (e.g., more than 400 mg of caffeine per day (more than 4 cups or glasses per day).

Interfering substance

25. Any medication (including St John's Wort) within 14 days before inclusion or within 5-times the elimination half-life (whichever is longer) of the medication.
26. Any vaccination within the last 28 days.
27. Any corticosteroids, anti-inflammatory drugs, immunomodulators or anticoagulants. Any participant currently receiving or having previously received immunosuppressive therapy, including systemic steroids including adrenocorticotrophic hormone (ACTH) or inhaled steroids in dosages which are associated with hypothalamic-pituitary-adrenal axis suppression such as 1 mg/kg/day of prednisone or its equivalent or chronic use of inhaled high potency corticosteroids (budesonide 800 µg per day or fluticasone 750 µg).
28. Any recent or current systemic therapy with an antibiotic or drug with potential antimalarial activity (chloroquine, piperaquine, benzodiazepine, flunarizine, fluoxetine, tetracycline, azithromycin, clindamycin, hydroxychloroquine, etc.).

General conditions

29. Any participant who, in the judgment of the Investigator, was likely to be noncompliant during the study, or unable to cooperate because of a language problem or poor mental development.
30. Any participant in the exclusion period of a previous study according to applicable regulations.
31. Any participant who lived alone (from Day 0 until at least the end of the antimalarial drug treatment).
32. Any participant who could not be contacted in case of emergency for the duration of the trial and up to 2 weeks following end of study visit.
33. Any participant who was the Investigator or any sub-investigator, research assistant, pharmacist, study coordinator, or other staff thereof, directly involved in conducting the study.
34. Any participant without a good peripheral venous access.

Biological status

35. Positive result on any of the following tests: hepatitis B surface (HBs Ag) antigen, anti-hepatitis B core antibodies (anti-HBc Ab), anti-hepatitis C virus (anti-HCV) antibodies, anti-human immunodeficiency virus 1 and 2 antibodies (anti-HIV1 and anti-HIV2 Ab).
36. Participant was found to be G6PD deficient.
37. Any drug listed in the protocol (Table 3) in the urine drug screen unless there is an explanation acceptable to the medical investigator (e.g., the participant stated in advance that they consumed a prescription or OTC product which contained the detected drug) and/or the participant had a negative urine drug screen on retest by the pathology laboratory.
38. Positive alcohol urine or breath test.

Specific to the study

39. Cardiac/QT risk:
• Known pre-existing prolongation of the QTcB/QTcF interval considered clinically significant.
• Family history of sudden death or of congenital prolongation of the QTc interval or known congenital prolongation of the QTc-interval or any clinical condition known to prolong the QTc interval. History of symptomatic cardiac arrhythmias or with clinically relevant bradycardia. Electrolyte disturbances, particularly hypokalaemia, hypocalcaemia or hypomagnesaemia.
• ECG abnormalities in the standard 12-lead ECG (at screening), which in the opinion of the Investigator was clinically relevant or would have interfered with the ECG analysis.
• A history of clinically significant ECG abnormalities.
40. Known hypersensitivity to MMV390048 or any of its excipients or 4-aminoquinolines, artemether or other artemisinin derivatives, lumefantrine, or other arylaminoalcohols.

41. Unwillingness to abstain from consumption of citrus (grapefruit, Seville orange, etc.) or their juices, as well as quinine containing foods/beverages such as tonic water, lemon bitter, from inoculation (Day 0) to the end of the malaria treatment.
42. Any history or presence of lactose intolerance.
43. Unwillingness to restrict exposure to direct sunlight during the study. Participants were advised to use sunglasses and a sunblock preparation for the remainder of the study period.

On dosing days

1. Ingestion of any drug since the recruitment interview (other than the doses administered in this study) that, in the opinion of the Investigator, could compromise the study.
2. Ingestion of any other drug, in the week prior to dosing or during the blood sampling period that, in the opinion of the Investigator, could compromise the study, e.g., through pharmacokinetic or metabolic interactions, or analytical interference. However the Investigator might permit the use of paracetamol for the treatment of headache or other pain. If drug therapy other than paracetamol, or drug specified in the protocol, is required during the study periods, a decision to continue or discontinue the participant’s participation was to be made by the Investigator, based on the nature of the medication and the time the medication was taken.
3. Failure to conform to the requirements of the protocol.
4. Detection of any drug listed in the study protocol in the urine drug screen unless there was an explanation acceptable to the Investigator (e.g., the participant had stated in advance that they consumed a prescription or OTC product which contained the detected drug).
5. Vital signs outside the reference range and clinically significant.

Inclusion/exclusion criteria for the formulation optimisation study

Inclusion Criteria

To be eligible for inclusion in this study, each subject fulfilled each of the following criteria:

1. Healthy male or female (of non-childbearing potential) aged 18 to 55 years (inclusive) at screening.
2. Had a body weight of at least 50 kg and a body mass index (BMI) of 18.0–30.0 Kg/m2 (inclusive).
3. Female subjects were of non-childbearing potential:
i. Natural (spontaneous) post-menopausal defined as being amenorrheic for at least 12 months without an alternative medical cause with a screening follicle stimulating hormone level >25 IU/L (or at the local laboratory levels for post-menopause).
ii. Premenopausal with irreversible surgical sterilization by hysterectomy and/or bilateral oophorectomy or salpingectomy at least 6 months before screening (as determined by subject medical history).
4. Male subjects agreed to use acceptable methods of contraception if the male subject's partner could become pregnant from the time of the first administration of study medication until 120 days following administration of the last dose of study medication. Subjects agreed to use one of the following acceptable methods of contraception:
i. Condom and occlusive cap (diaphragm or cervical/vault caps) used with spermicidal foam/gel/film/cream/suppository.
ii. Surgical sterilization (vasectomy with documentation of azoospermia) and a barrier method (condom or occlusive cap [diaphragm or cervical/vault caps] used with spermicidal foam/gel/film/cream/suppository).
iii. The female partner used oral contraceptives (combination estrogen/progesterone pills), injectable progesterone or subdermal implants and a barrier method (condom or occlusive cap [diaphragm or cervical/vault caps] used with spermicidal foam/gel/film/cream/suppository).
iv. The female partner used medically prescribed topically-applied transdermal contraceptive patch and a barrier method (condom or occlusive cap [diaphragm or cervical/vault caps] used with spermicidal foam/gel/film/cream/suppository).
v. The female partner had undergone documented tubal ligation (female sterilization). In addition, a barrier method (condom or occlusive cap [diaphragm or cervical/vault caps] used with spermicidal foam/gel/film/cream/suppository) must be used.
vi. The female partner had undergone documented placement of an intrauterine device or intrauterine system. In addition, a barrier method (condom or occlusive cap [diaphragm or cervical/vault caps] used with spermicidal foam/gel/film/cream/suppository) must be used.
vii. True abstinence: when this was in line with the preferred and usual lifestyle of the subject. Periodic abstinence (e.g. calendar, ovulation, symptothermal, post-ovulation methods) and withdrawal were not acceptable methods of contraception. Abstinent subjects agreed to use one of the above-mentioned contraceptive methods, if they started sexual relationships during the study and for up to 120 days after the last dose of study drug.
5. Subjects were non-smokers or ex-smokers for more than 90 days prior to screening or smoked no more than 5 cigarettes per day. If users of nicotine products (i.e. spray, patch, e-cigarette, etc.) used the equivalent to no more than 5 cigarettes per day.
6. Subjects were not to donate egg or sperm from the time of the first administration of treatment or study medication until 120 days following administration of the last treatment or dose of study medication.

7. Subjects were able to understand and comply with the requirements of the study and had signed the informed consent form prior to undergoing any study-related procedures.
8. Subjects agreed to avoid excessive UV radiation exposure (i.e., occupational exposure to the sun, sunbathing, tanning salon use, phototherapy, etc.) throughout the study.

Exclusion Criteria

A subject was not eligible for inclusion in this study if any of the following criteria applied:

1. Male subjects with a female partner(s) who was (were) pregnant or lactating from the time of the administration of study medication.
2. Women were of childbearing potential, defined as all women physiologically capable of becoming pregnant, including women whose career, lifestyle, or sexual orientation precludes intercourse with a male partner and women whose partners have been sterilized by vasectomy or other means.
3. Current or recurrent disease (e.g. cardiovascular, neurological, renal, gastrointestinal, oncologic or other conditions) that affects the action, absorption or disposition of the study medication or affects clinical assessments or clinical laboratory evaluations.
4. Current or relevant history of physical or psychiatric illness that required treatment or made the subject unlikely to fully comply with the requirements or complete the study, or any condition that presented undue risk from the investigational product or study procedures.
5. Any other significant disease or disorder which, in the opinion of the Investigator, either put the subject at risk because of the participation in the study, influenced the result of the study, or the subject's ability to participate in the study.
6. History of photosensitivity.
7. History or clinical evidence of alcohol or substance abuse. Alcohol abuse is defined as regular weekly intake of more than 21 units for males and 14 units for females.
8. Any clinically relevant history of intolerance/allergy to milk or dairy products.
9. Use of an investigational product or participation in a clinical study within 90 days prior for study medication administration.
10.Donation of blood products (e.g. plasma or platelets) or of more than 500 ml of blood within 90 days prior to study medication administration.
11.Use of any prescription drugs within 14 days or within 5 times the elimination half-life (whichever period was longer) prior to study medication administration.
12.Used moderate or strong inhibitors and/or inducers of CYP450/Transporters within 4 weeks prior to the planned drug administration (or at least 5 half-lives of the compound whichever period was the longer).
13.Use of over-the-counter (OTC) medications or dietary supplements, including vitamins and herbal supplements within 7 days of the study medication administration. With the exception of paracetamol which may be used incidentally or for a short-term treatment at a maximum dose of 2 gr. per day.
14. Intake of grapefruit, grapefruit juice or other products containing grapefruit within 28 days prior of the first administration of the study drug.
15.Excessive intake of caffeine drinks or energy drinks within 48 hours before admission defined as more than three 250 ml cups of coffee a day, equivalent to roughly 250 mg of caffeine.

16.Any clinically significant abnormal laboratory, vital signs or other safety findings as determined by medical history, physical examination or other evaluations conducted at screening or on admission.

17.Any liver function tests elevated >1.5 times the upper limit of normal (ULN), considered by the Investigator as clinically relevant, at screening or admission visits.

18.Abnormal serum haemoglobin, haptoglobin, reticulocyte count and lactate dehydrogenase (LDH) at screening or admission.

19.Subject with abnormal ECG results at the screening or admission visit and these results were considered as clinically significant by the Investigator.

20.Confirmed positive results from urine drug screen (amphetamines, benzodiazepines, cocaine, cannabinoids, opiates, barbiturates, and methadone) or from the alcohol breath test at screening and on admission.

21.A positive human immunodeficiency virus (HIV) I and II antibodies, hepatitis B surface antigen (HBsAg), anti Hepatitis core antibody (anti HBc Ig G [and anti HBc IgM if IgG is positive], or hepatitis C virus (HCV) antibody at screening.

22.Subjects had veins unsuitable for intravenous puncture or cannulation on either arm (e.g. veins that are difficult to locate access or puncture veins with a tendency to rupture during or after puncture).

23.Any conditions which in the opinion of the Investigator would make the subject unsuitable for enrolment or would interfere with the subjects’ participation in or completion of the study.

Schedule of events for each clinical trial

Table S1: Schedule of events for the first-in-human study

Trial Phase	Screening [a]	Baseline	Treatment and Follow-up																			Trial Completion
Visit Numbers	1	2	3													4	5	6	7	8	9	10
Trial days	Day -28 to -2	Day -1	1											2	3	5	7	10	14	19	26	29
Time (h)		-24	Pre-dose	0	0.5	1	2	3	4	6	8	9	12	24	48	96	144	216	312	432	600	672
Informed Consent	X																					
Admission to unit		X																				
Meal record [b]		X---	---	---	---	---	---	---	---	---	---	---	X---	X								
Inclusion & Exclusion criteria	X	X	X																			
Medical history / current medical conditions	X	X																				
Demography	X																					
Physical examination	X	X[c]	X[c]												X[c]	X[c]	X[c]	X[c]	X	X[c]	X[c]	X[c]
Hepatitis B & C, HIV screen	X																					
Alcohol test, drug screen & serum cotinine	X	X																				
Pregnancy test (females)	X	X																				X
Endocrinology assessments (males)[d]	X	X															X		X			
Body height	X																					
Body weight	X	X															X		X			X
Body temperature	X	X	X			X			X			X			X							X
Blood Pressure & Heart Rate	X[e]	X	X		X	X	X	X	X	X	X		X	X	X	X	X	X	X	X	X	X
ECG	X	X	X			X	X	X	X	X	X			X	X	X	X					
Telemetry		X[f]	X---	---	---	---	---	---	---	---	---	---	---	X								
Safety haematology, coagulation & biochemistry	X	X	X[g]											X	X	X	X	X	X		X	X
Urinalysis (dipstick)	X		X											X[h]	X[h]				X			X
Peripheral blood smear microscopy	X	X													X		X		X			
Haemolysis panel	X	X												X[i]	X[i]	X[i]	X	X	X	X[i]		X
LDH														X[j]	X[j]	X[j]	X[j]	X[j]	X[j]	X[j]		X[j]
Study Drug Administration				X																		
Malaria bioassay blood collection [k]					X	X	X	X	X	X	X	X	X	X	X	X						
PK blood collection [l]			X		X	X	X	X	X	X	X		X	X	X	X	X	X	X	X	X	X
Urine PK collection [m]			X	X---	---	---	---	---	---	---	---	---	X---	X---	X							
Pharmacogenetics [n]			X																			
Discharge from unit															X		X[o]					

Trial Phase	Screening [a]	Baseline	Treatment and Follow-up																			Trial Completion
Visit Numbers	1	2	3													4	5	6	7	8	9	10
Trial days	Day -28 to -2	Day -1	1											2	3	5	7	10	14	19	26	29
Time (h)		-24	Pre-dose	0	0.5	1	2	3	4	6	8	9	12	24	48	96	144	216	312	432	600	672
Adverse Events				As required																		
Serious Adverse Events				As required to 30 days post trial completion																		
Concomitant meds/ Therapies	X	X	X	As required																		

a Subjects in Cohort SAD7 (Fed) will not be re-screened
b Meal records will be collected for the duration of admission viz., Day -1 to Day 5 for Cohort SAD1 and Day -1 to Day 3 for Cohorts SAD2-8
c Abbreviated (symptom-directed) physical exam only to be conducted as relevant
d FSH, LH, testosterone and Inhibin B (serum sample for measurement of Inhibin B will be stored for later exploratory analysis)
e Supine & standing blood pressure and pulse measurements required at screening
f 24-hour baseline telemetry monitoring will be performed at any stage during the screening/baseline period
g Pre-dose fasting glucose only
h Urine to be collected from first void mornings Days 2 & 3
i Excluding DAT, all other time points full panel required
j LDH with isoenzymes (LDH1 and LDH2) if total LDH > ULN after treatment
k Cohort 3 only
l Additional PK data points may be planned beyond the planned final visit depending on emerging PK data; unnecessary PK data points could be eliminated for the latter cohorts
m Cohorts 4 to 7 only
n Pharmacogenetic sample optional, collected from consented subjects ONLY
o Cohort 1 only will remain admitted until discharge on Day 5

Table S2: Schedule of events for the IBSM study

Procedures	Screen	Challenge Inoculum		Malaria Monitoring		MMV390048 Treatment	Safety monitoring	Riamet® Treatment	Safety Monitoring	Final Visit or EOS
**Day**	**-d28 to –d3**	**0**	**1,2 &3**	**4AM until admission**	**Admi-ssion**	**Confinement (study day 7-10)**	**Up to 21 days post treatment**	**Timing as per protocol**	**Study day Riamet® (+48h)**	**Day 28±3**
Informed consent & eligibility	X									
Medical History	X									
Physical Examination [a]	X	X		X	X	X	X	X	X	X
ECG [b]	X	X			X	X		X		X
Vital Signs [c]	X	X		X	X	X	X	X	X	X
Haem & Biochem [d]	X	X			X	X	X[d]	X	X	X
Serology [e]	X									X
Red Cell Allo-Antibody	X									X
Urinalysis [f]	X									X
Urine Drug and Alcohol Screen [g]	X	X			X					
**Blood stage challenge**		X								
**Phone Call**			X						X[j]	
Unit Confinement [h]					X	X				
**Drug Treatment**						X[i]		X[j]	X[j]	
**Adverse Event**		X	X	X	X	X	X	X	X	X
Malaria qPCR [k]		X		X[k]	X	X	X[k]	X[k]	X[k]	X[k]
MMV390048 Drug level [l]						X[l]	X[l]			X[l]

Safety Serum Storage		X								X

[a] A complete physical examination was to be performed at screening and the final visit (EOS). An abbreviated physical examination was to be performed upon admission to the unit and at all morning and evening visits during confinement and where symptoms of malaria were identified.

[b] ECG – 12-lead electrocardiogram were to be recorded in triplicate at screening, Day 0 pre malaria inoculum, on entry for confinement predose MMV390048, post MMV390048 treatment (2, 4 and 24 h post dose), prior to exit from clinical unit (72 h post dose), pre Riamet® treatment, and at Day 28 or End Of Study.

[c] Temperature (sublingual), respiratory rate, heart rate and blood pressure were to be obtained at screening, pre dose malaria inoculum, and at approximately 60 min post inoculum (prior to leaving the clinical unit). Also these vital signs were to be measured at each outpatient visit and at morning, midday and evening during confinement, or when malaria symptoms were presented. Participants were to remain semi-recumbent for 10 minutes prior to measurement of heart rate and blood pressure.

[d] See Protocol for specific haematology and chemistry tests. Liver function test was to be performed 5 days post MMV390048.

[e] Viral and other Serology: HIV, Hepatitis B, Hepatitis C, EBV, CMV.

[f] Urinalysis was to be performed at screening and at Day28 or EOS only.

[g] Drug screen, alcohol breath test: screening, Day 0 pre malaria inoculum, and on admission to the unit for MMV390048 dosing.

[h] Confinement period 72 h (planned to be approximately from Day 7 AM to Day 10 AM).

[i] MMV390048 treatment: individual participant treatment was to happen within 24 h if PCR parasitaemia was ≥5000 parasites/mL and asymptomatic, or cohort treatment if PCR parasitaemia in all participants was ≥1000 parasites/mL.

j Riamet®: 3 days until completion of Riamet® treatment (0, 12, 24, 36, 48, 60 hour). Phone calls were to be made to the participants to check on symptoms and ensure completion with treatment. Some doses were to be taken on site, others at home with phone calls to confirm dosing.

[k] qPCR: Day 0, then AM testing from Day 4 AM until PCR positive for malaria, and then AM or AM and PM from Day 5 till confinement. Following confinement on noted days until the last Riamet® dosing day, and at the final visit (Day 28/EOS). qPCR during confinement: 0, 2, 4, 8, 12, 16, 20, 24, 30, 36, 48, 60 and 72 hours post MMV390048 dosing.

[l] Blood for pharmacokinetic assessment of MMV390048 at T(0) = pre dose, 0.5, 1, 2, 3, 4, 6, 8, 12, 14, 16, 20, 24 (MMV390048+Day 1), 30, 48 (MMV390048+Day 2), 72 (MMV390048+Day 3), 96 (MMV390048+Day 4), and 144 h (MMV390048+Day 6) post MMV390048 dose, and Days +8, 10, 14, 18 and 21 post MMV390048 dose.

Table S3: Schedule of events for the formulation optimisation study

Clinical evaluations from screening to follow-up (Day 29)

Study Phase	Screening	Baseline	Treatment											Follow-up						Study completion
Visit numbers	1	2	3											4	5	6	7	8	9	10
Study Days	-28 to -2	-1	1									2	3	5	7	10	14	19	26/ ET[n]	29
Time (h)		-24	Pre-dose	0	1	2	3	4	6	8	12	24	48	96	144	216	312	432	600	672
Written informed consent form [a]	X																			
Inclusion/Exclusion criteria verification	X	X	X																	
Medical history/Current medical conditions	X	X																		
Demographics	X																			
Body height	X[b]																			
Body weight	X[b]	X																		X
Physical examination [c]	X	X	X										X							X
Alcohol breath test and drug screen	X	X																		
Pregnancy test [d]	X																			
Follicle stimulating hormone, FSH [e]	X																			
Vital signs [f]	X	X	X			X		X		X		X	X						X	X
Body temperature [g]	X	X																	X	
12 lead ECG [h]	X	X	X		X	X		X		X	X	X	X						X	X
Safety haematology, coagulation and biochemistry[i]	X	X										X	X						X	
Serology	X																			
Urinalysis	X	X										X	X						X	
Study Drug Administration				X																
PK blood collection			X		X	X	X	X	X	X	X	X	X	X	X	X	X	X	X	X
Meal record [j]		X[k]						X →				X	X							
Adverse events [l]				X →																X

**Study Phase**	Screening	Baseline	Treatment												Follow-up						Study completion
**Visit numbers**	1	2	3											4	5	6	7	8	9	10
**Study Days**	-28 to -2	-1	1									2	3	5	7	10	14	19	26/ ET[n]	29
**Time (h)**		-24	Pre-dose	0	1	2	3	4	6	8	12	24	48	96	144	216	312	432	600	672
**Serious Adverse Events** [m]				X →																X
**Concomitant medications**	X	X	X →																	X
**Admission to Unit**		X																		
**Discharge from unit**													X							

[a] Written informed consent was obtained before performing any procedures.
[b] BMI was performed at screening.
[c] Full physical examination was performed at screening. A brief physical examination was performed on admission (Day -1), Day 1 pre-dose, 48 hours post-dose and on Day 29 post-dose focusing on any changes since the preceding full examination.
[d] Serum pregnancy testing was not performed at screening. The study enrolled only male volunteers.
[e] FSH (follicle stimulating hormone) testing was not performed at screening. The study enrolled only male volunteers.
[f] Vital signs included blood pressure (supine) and heart rate which were performed at screening, on admission (Day -1), Day 1 pre-dose, 2, 4, 8, 24 and 48 hours post-dose and on Days 26 and 29 post-dose.
[g] Body temperature was measured using tympanic thermometers and was performed at screening, on admission (Day -1) and on Day 26 post-dose.
[h] Triplicate 12-lead ECG after the subjects were resting in a supine position for at least 10 minutes were performed at screening, on admission (Day -1), Day 1 pre-dose, 1, 2, 4, 8, 12, 24 and 48 hours post-dose and on Days 26 and 29 post-dose.
[I] Safety blood samples included LDH, reticulocyte count and haptoglobin.
[j] Subjects fasted from 8 hours prior to dosing. They had their first meal 4 hours post-dose on dosing day; all other meals were served at standard unit times.
[k] The optional cohort was not conducted.
[l] AE were collected from dosing until study completion (last visit), any changes in health occurring after signing informed consent but prior to dosing were recorded in medical history.
[m] SAE were collected until 30 days after last visit (SAEs recorded after study completion was spontaneously reported by the subject).
[n] In case of Early Termination (ET), the assessments performed on Day 26 were conducted.

Assay for measuring MMV390048 concentrations in human plasma

Determination of MMV390048 from 50 µL human plasma by LC-MS/MS

A method was validated for the quantification of MMV390048 in human plasma, and consisted of a protein precipitation extraction, followed by high performance liquid chromatography with MS/MS detection. MMV390048-d3 was used as the internal standard. Analyte mean extraction yield was approximately 106.5 % (CV% = 6.2).

The extraction procedure was followed by liquid chromatographic separation using a Waters Atlantis T3 3 µm, 100 x 2.1 mm analytical column. An isocratic mobile phase containing water:acetonitrile (25:75 [v/v]) was used at a flow-rate of 300 µL/min. The retention times for MMV390048 and MMV390048-d3 were 1.16 min and 1.16 min, respectively. An AB Sciex API 4000 mass spectrometer at unit resolution in the multiple reaction monitoring mode was used to monitor the transition of the protonated precursor ions, 394.145 m/z and 397.148 m/z, to the product ions, 297.0 m/z and 297.0 m/z, for MMV390048 and the internal standard, respectively. Electro-spray ionisation was used for ion production.

Accuracy and precision were assessed over three consecutive, independent runs. The calibration curve fits a quadratic (weighted by 1/x) regressions for MMV390048 over the ranges 0.500 – 2000 ng/mL, based on peak area ratios. The assay was successfully cross-validated on a second/back-up API 4000 instrument. A 1:4 dilution of the QC dilution sample showed that concentrations of up to 4000 ng/mL of MMV390048 in plasma could be analysed reliably when diluted into the calibration range. No carry-over of MMV390048 was observed with the Agilent autosampler. Endogenous matrix components were found to have an insignificant effect on the reproducibility of the method when human plasma originating from six different sources were analysed.

MMV390048 was found to be stable in human plasma for up to 79 days when stored at approximately -80°C, for 6 hours at room temperature, and when subjected to 3 freeze-thaw cycles. Stock solutions of MMV390048 in dimethylsulfoxide was stable for 67 days when stored at -80°C and for 24 hours when stored at room temperature, 4°C and -20°C. Plasma extracts of the analyte/internal standard were shown to be stable on-instrument, over a period of 40 hours. Reinjection reproducibility experiments indicated that an assay batch may be re-injected after 26 hours. MMV390048 was found to be stable in whole blood (on ice) for up to 1 hour.

Quantification of MMV390048 in plasma was not significantly affected by the presence of 1% haemolysed blood at both high and low concentrations. Similarly, 2% and 10% haemolysis did not significantly affect quantitation at high plasma concentrations. At low plasma concentrations (1.00 ng/mL) assay results indicates that 2% and 10% haemolysis has an effect on the precision of the assay but accuracy was still within the acceptance criteria. Samples with haemolysis greater than 1% were assayed in duplicate to confirm results.

Adverse events by MedDRA system organ class and preferred term for each clinical trial

Table S4: Adverse events for the first-in-human study

All adverse events by treatment group
Population: Safety

MedDRA system organ class/ Preferred term for adverse event	Treatment group						
	MMV390048						
	5mg	20mg	40mg	80mg	120mg	40mg FED	Placebo
	n (m) %	n (m) %	n (m) %	n (m) %	n (m) %	n (m) %	n (m) %
Number of participants	6	6	6	6	6	6	12
Number of participants with at least one AE	4 (11) 66.7	5 (12) 83.3	5 (19) 83.3	6 (20) 100.0	6 (11) 100.0	6 (22) 100.0	11 (32) 91.7
General disorders and administration site conditions	3 (3) 50.0	3 (3) 50.0	3 (3) 50.0	5 (8) 83.3	3 (3) 50.0	5 (6) 83.3	8 (12) 66.7
Medical device site reaction	3 (3) 50.0	0	1 (1) 16.7	5 (5) 83.3	3 (3) 50.0	5 (5) 83.3	5 (5) 41.7
Influenza like illness	0	2 (2) 33.3	2 (2) 33.3	2 (2) 33.3	0	1 (1) 16.7	2 (3) 16.7
Fatigue	0	0	0	0	0	0	2 (2) 16.7
Catheter site pain	0	0	0	0	0	0	1 (1) 8.3
Chest pain	0	0	0	0	0	0	1 (1) 8.3
Malaise	0	0	0	1 (1) 16.7	0	0	0
Vessel puncture site haematoma	0	1 (1) 16.7	0	0	0	0	0
Gastrointestinal disorders	0	1 (1) 16.7	3 (3) 50.0	2 (2) 33.3	0	3 (4) 50.0	5 (7) 41.7
Diarrhoea	0	1 (1) 16.7	1 (1) 16.7	1 (1) 16.7	0	2 (2) 33.3	1 (1) 8.3
Constipation	0	0	1 (1) 16.7	1 (1) 16.7	0	0	1 (1) 8.3
Abdominal pain	0	0	0	0	0	0	1 (1) 8.3
Dyspepsia	0	0	1 (1) 16.7	0	0	0	0

AE - adverse event.
n = number of participants, (m) = number of mentions.
Percentages are expressed as the percentage of the number of participants for each treatment group.
Adverse event data were coded using MedDRA version 17.1.

All adverse events by treatment group
Population: Safety

MedDRA system organ class/ Preferred term for adverse event	Treatment group: MMV390048 5mg n	(m)	%	20mg n	(m)	%	40mg n	(m)	%	80mg n	(m)	%	120mg n	(m)	%	40mg FED n	(m)	%	Placebo n	(m)	%
Gastrointestinal motility disorder	0			0			0			0			0			0			1	(1)	8.3
Gastrooesophageal reflux disease	0			0			0			0			0			1	(1)	16.7	0		
Haemorrhoids	0			0			0			0			0			0			1	(1)	8.3
Nausea	0			0			0			0			0			0			1	(1)	8.3
Toothache	0			0			0			0			0			0			1	(1)	8.3
Vomiting	0			0			0			0			0			1	(1)	16.7	0		
Nervous system disorders	0			1	(1)	16.7	4	(6)	66.7	3	(4)	50.0	2	(3)	33.3	1	(1)	16.7	1	(1)	8.3
Headache	0			1	(1)	16.7	2	(3)	33.3	1	(2)	16.7	1	(1)	16.7	1	(1)	16.7	1	(1)	8.3
Dizziness	0			0			2	(2)	33.3	0			0			0			0		
Dysgeusia	0			0			0			1	(1)	16.7	0			0			0		
Generalised tonic-clonic seizure	0			0			0			0			1	(1)	16.7	0			0		
Hypoaesthesia	0			0			1	(1)	16.7	0			0			0			0		
Myoclonus	0			0			0			0			1	(1)	16.7	0			0		
Tension headache	0			0			0			1	(1)	16.7	0			0			0		
Infections and infestations	1	(1)	16.7	2	(2)	33.3	2	(2)	33.3	1	(3)	16.7	1	(1)	16.7	2	(2)	33.3	1	(1)	8.3

AE - adverse event.
n = number of participants, (m) = number of mentions.
Percentages are expressed as the percentage of the number of participants for each treatment group.
Adverse event data were coded using MedDRA version 17.1.

All adverse events by treatment group
Population: Safety

MedDRA system organ class/ Preferred term for adverse event	5mg n	(m)	%	20mg n	(m)	%	40mg n	(m)	%	80mg n	(m)	%	120mg n	(m)	%	40mg FED n	(m)	%	Placebo n	(m)	%
Upper respiratory tract infection	0			1	(1)	16.7	1	(1)	16.7	1	(1)	16.7	0			0			0		
Furuncle	1	(1)	16.7	0			0			1	(2)	16.7	0			0			0		
Urinary tract infection	0			1	(1)	16.7	0			0			0			0			1	(1)	8.3
Hordeolum	0			0			1	(1)	16.7	0			0			0			0		
Oral herpes	0			0			0			0			0			1	(1)	16.7	0		
Sexually transmitted disease	0			0			0			0			0			1	(1)	16.7	0		
Tinea versicolour	0			0			0			0			1	(1)	16.7	0			0		
Investigations	0			2	(2)	33.3	3	(4)	50.0	1	(1)	16.7	2	(2)	33.3	1	(1)	16.7	1	(1)	8.3
Blood creatine phosphokinase increased	0			0			3	(3)	50.0	1	(1)	16.7	2	(2)	33.3	0			0		
Neutrophil count decreased	0			0			1	(1)	16.7	0			0			1	(1)	16.7	0		
Blood pressure decreased	0			1	(1)	16.7	0			0			0			0			0		
Electrocardiogram PR prolongation	0			1	(1)	16.7	0			0			0			0			0		
Haemoglobin decreased	0			0			0			0			0			0			1	(1)	8.3
Injury, poisoning and procedural complications	4	(5)	66.7	0			0			0			0			3	(3)	50.0	1	(4)	8.3
Contusion	3	(3)	50.0	0			0			0			0			0			0		

Treatment group: MMV390048 (5mg, 20mg, 40mg, 80mg, 120mg, 40mg FED); Placebo

AE - adverse event.
n = number of participants, (m) = number of mentions.
Percentages are expressed as the percentage of the number of participants for each treatment group.
Adverse event data were coded using MedDRA version 17.1.

All adverse events by treatment group
Population: Safety

MedDRA system organ class/ Preferred term for adverse event	MMV390048 5mg n (m) %	MMV390048 20mg n (m) %	MMV390048 40mg n (m) %	MMV390048 80mg n (m) %	MMV390048 120mg n (m) %	MMV390048 40mg FED n (m) %	Placebo n (m) %
Thermal burn	0	0	0	0	0	2 (2) 33.3	0
Animal bite	0	0	0	0	0	0	1 (2) 8.3
Animal scratch	0	0	0	0	0	0	1 (1) 8.3
Excoriation	1 (1) 16.7	0	0	0	0	0	0
Human bite	1 (1) 16.7	0	0	0	0	0	0
Laceration	0	0	0	0	0	0	1 (1) 8.3
Skin abrasion	0	0	0	0	0	1 (1) 16.7	0
Skin and subcutaneous tissue disorders	1 (1) 16.7	2 (2) 33.3	0	0	0	1 (2) 16.7	1 (1) 8.3
Urticaria	0	1 (1) 16.7	0	0	0	1 (1) 16.7	0
Dermatitis bullous	0	0	0	0	0	0	1 (1) 8.3
Miliaria	0	0	0	0	0	1 (1) 16.7	0
Night sweats	1 (1) 16.7	0	0	0	0	0	0
Urticaria papular	0	1 (1) 16.7	0	0	0	0	0
Eye disorders	0	0	0	1 (1) 16.7	1 (1) 16.7	1 (1) 16.7	1 (1) 8.3
Conjunctivitis allergic	0	0	0	0	0	0	1 (1) 8.3

AE - adverse event.
n = number of participants, (m) = number of mentions.
Percentages are expressed as the percentage of the number of participants for each treatment group.
Adverse event data were coded using MedDRA version 17.1.

All adverse events by treatment group
Population: Safety

MedDRA system organ class/ Preferred term for adverse event	Treatment group: MMV390048 5mg n	(m)	%	20mg n	(m)	%	40mg n	(m)	%	80mg n	(m)	%	120mg n	(m)	%	40mg FED n	(m)	%	Placebo n	(m)	%
Eye pruritus	0			0			0			0			1	(1)	16.7	0			0		
Photophobia	0			0			0			1	(1)	16.7	0			0			0		
Pterygium	0			0			0			0			0			1	(1)	16.7	0		
Respiratory, thoracic and mediastinal disorders	1	(1)	16.7	1	(1)	16.7	0			1	(1)	16.7	1	(1)	16.7	0			0		
Oropharyngeal pain	1	(1)	16.7	1	(1)	16.7	0			0			0			0			0		
Epistaxis	0			0			0			1	(1)	16.7	0			0			0		
Sneezing	0			0			0			0			1	(1)	16.7	0			0		
Musculoskeletal and connective tissue disorders	0			0			0			0			0			2	(2)	33.3	1	(1)	8.3
Back pain	0			0			0			0			0			1	(1)	16.7	1	(1)	8.3
Myalgia	0			0			0			0			0			1	(1)	16.7	0		
Cardiac disorders	0			0			1	(1)	16.7	0			0			0			1	(1)	8.3
Palpitations	0			0			1	(1)	16.7	0			0			0			1	(1)	8.3
Reproductive system and breast disorders	0			0			0			0			0			0			1	(2)	8.3
Postmenopausal haemorrhage	0			0			0			0			0			0			1	(1)	8.3
Vaginal discharge	0			0			0			0			0			0			1	(1)	8.3

AE - adverse event.
n = number of participants, (m) = number of mentions.
Percentages are expressed as the percentage of the number of participants for each treatment group.
Adverse event data were coded using MedDRA version 17.1.

Table S5: Adverse events for the IBSM study

All adverse events (Safety population)

System organ class	Preferred term	MMV390048 20 mg (N=6)	
		n (%)	M
Subjects with at least one AE		5 (83.3%)	32
Eye disorders		1 (16.7%)	1
	Eye swelling	1 (16.7%)	1
Gastrointestinal disorders		1 (16.7%)	1
	Abdominal discomfort	1 (16.7%)	1
	Nausea	2 (33.3%)	2
General disorders and administration site conditions		4 (66.7%)	11
	Chills	1 (16.7%)	2
	Hot flush	1 (16.7%)	1
	Hyperhidrosis	1 (16.7%)	1
	Pyrexia	3 (50.0%)	7
Metabolism and nutrition disorders		2 (33.3%)	2
	Decreased appetite	2 (33.3%)	2
Musculoskeletal and connective tissue disorders		1 (16.7%)	1
	Back pain	1 (16.7%)	1
	Muscle spasms	1 (16.7%)	1
	Myalgia	2 (33.3%)	2
Nervous system disorders		4 (66.7%)	7
	Headache	4 (66.7%)	7
	Paraesthesia	1 (16.7%)	1
Respiratory, thoracic and mediastinal disorders		1 (16.7%)	1
	Rinorrhea	1 (16.7%)	1
Skin and subcutaneous tissue disorders		1 (16.7%)	1
	Rash	1 (16.7%)	1
Vascular disorders		1 (16.7%)	1
	Contusion	1 (16.7%)	1

AE=adverse event
N=number of participants, M=number of mentions.
Adverse events were coded using MedDRA version 17.1

Table S6: Adverse events for the formulation optimisation study

Adverse Events - Frequency Table by Treatment (Safety Set)

System Organ Class*	Preferred Term**	Formulation A N=9 n (%) E	Formulation B N=9 n (%) E	Overall N=18 n (%) E
Total	Total	4 (44.4) 4	3 (33.3) 7	7 (38.9) 11
Gastrointestinal Disorders	Total	1 (11.1) 1	1 (11.1) 1	2 (11.1) 2
	Constipation	0 (0.0) 0	1 (11.1) 1	1 (5.6) 1
	Dyspepsia	1 (11.1) 1	0 (0.0) 0	1 (5.6) 1
Infections And Infestations	Total	2 (22.2) 2	1 (11.1) 1	3 (16.7) 3
	Upper Respiratory Tract Infection	0 (0.0) 0	1 (11.1) 1	1 (5.6) 1
	Viral Upper Respiratory Tract Infection	2 (22.2) 2	0 (0.0) 0	2 (11.1) 2
Injury, Poisoning And Procedural Complications	Total	0 (0.0) 0	2 (22.2) 2	2 (11.1) 2
	Injury	0 (0.0) 0	1 (11.1) 1	1 (5.6) 1
	Ligament Sprain	0 (0.0) 0	1 (11.1) 1	1 (5.6) 1
Nervous System Disorders	Total	0 (0.0) 0	2 (22.2) 2	2 (11.1) 2
	Headache	0 (0.0) 0	2 (22.2) 2	2 (11.1) 2
Respiratory, Thoracic And Mediastinal Disorders	Total	1 (11.1) 1	0 (0.0) 0	1 (5.6) 1
	Nasal Congestion	1 (11.1) 1	0 (0.0) 0	1 (5.6) 1
Skin And Subcutaneous Tissue Disorders	Total	0 (0.0) 0	1 (11.1) 1	1 (5.6) 1
	Rash	0 (0.0) 0	1 (11.1) 1	1 (5.6) 1

Abbreviation(s): N - Number of subjects at risk,
n - Number of subjects having an adverse event,
E - Number of events

Note(s): % - n/N*100
System Organ Class and Preferred Term according to MedDra dictionary Version 17.1 or higher.
*Multiple occurrences within a System Organ Class by a subject were counted once per System Organ Class.
**Multiple occurrences of a Preferred Term by a subject were counted once per Preferred Term.

Endocrinology results

Figure S1: Median concentration of follicle stimulating hormone, luteinizing hormone, testosterone and inhibin B over time by treatment group for male subjects

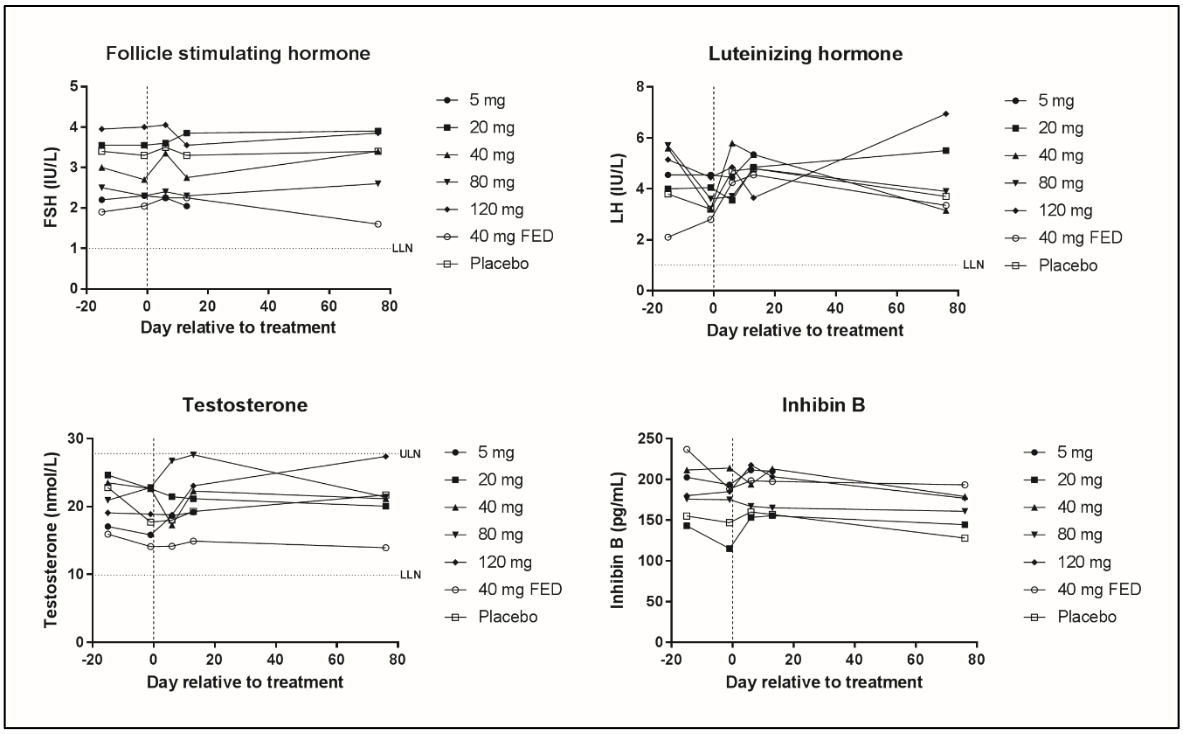

Plots represent the median concentration for male subjects within each treatment group. The screening time point occurred within 28 days prior to dosing and was different between subjects; for the purpose of graphing this time point was standardized to 15 days prior to dosing. The vertical dashed line represents the day of treatment with MMV390048 or placebo. LLN: lower limit of normal; ULN: upper limit of normal; FSH: follicle stimulating hormone; LH: luteinizing hormone.

Individual subject MMV390048 plasma concentration results

Figure S2: Individual subject MMV390048 plasma concentration-time profiles for the first-in-human study

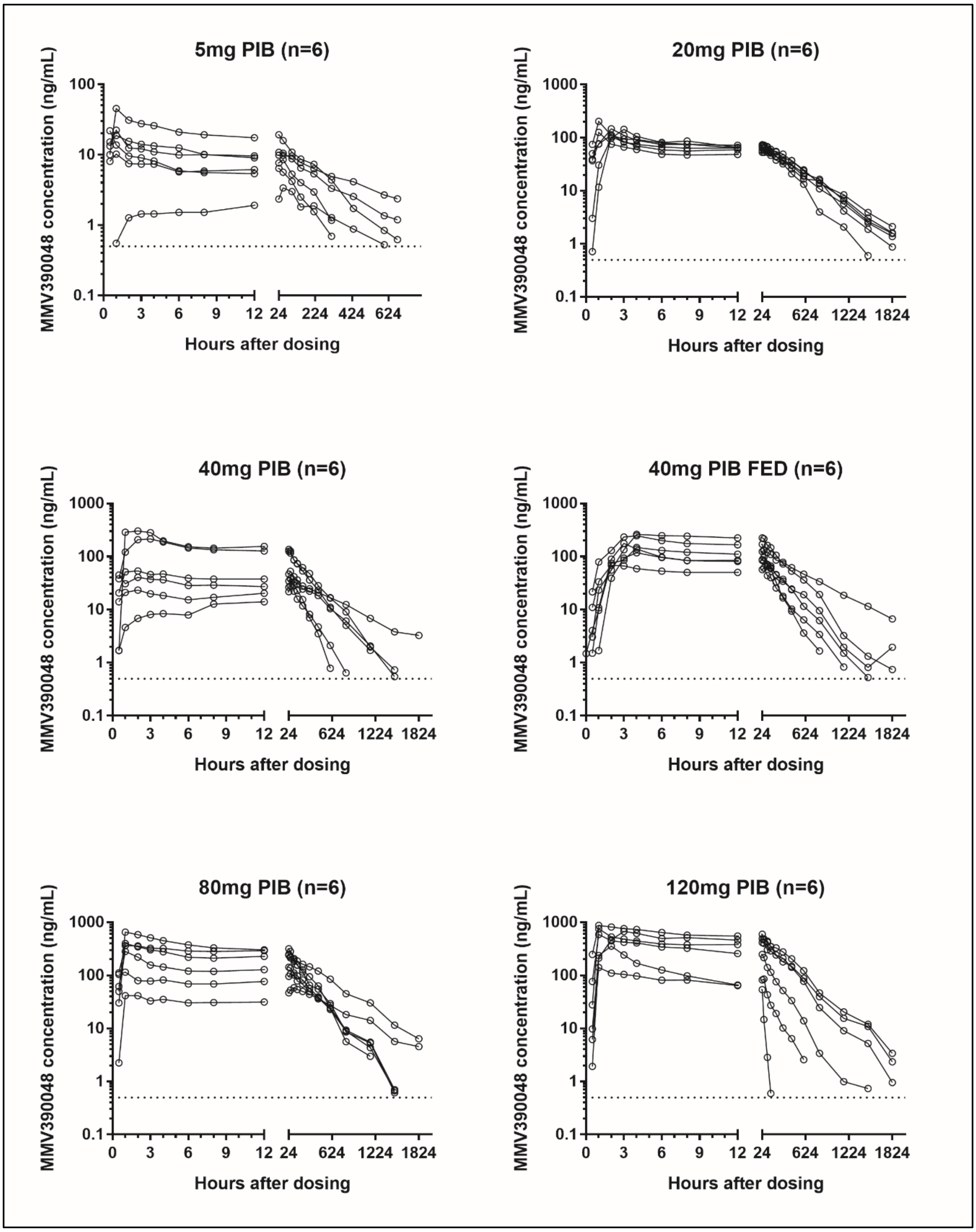

Plots represent the individual subject concentration-time profiles by dose cohort. Subjects dosed with placebo are excluded. The horizontal dotted lines represent the lower limit of quantitation (LLQ) of the assay (0.5 ng mL^{-1}). Points that are below the LLQ are excluded. PIB: powder-in-bottle formulation.

Figure S3: Individual subject MMV390048 plasma concentration-time profiles for the IBSM and formulation optimisation studies

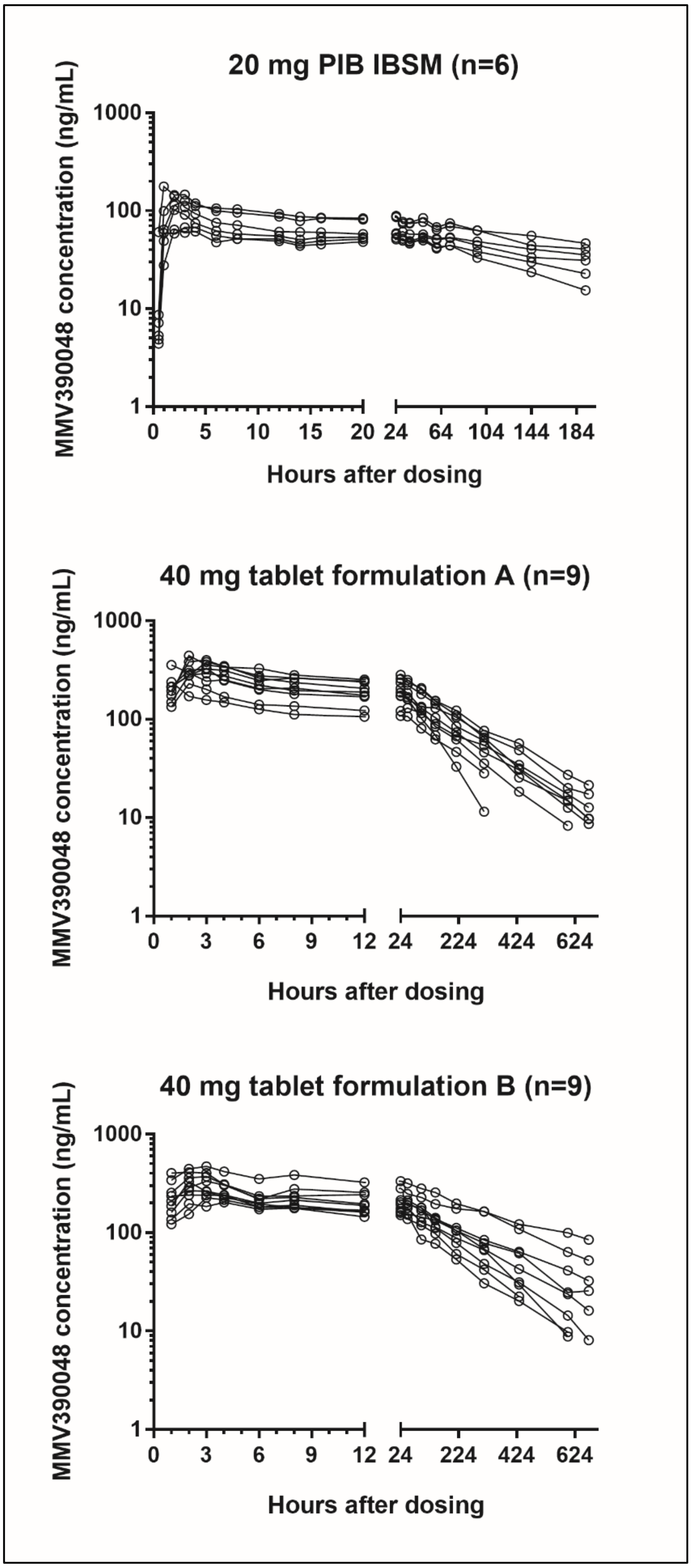

Plots represent the individual subject concentration-time profiles by dose cohort. Points that are below the LLQ are excluded. PIB: powder-in-bottle formulation; IBSM: induced blood stage malaria study; formulation A: tartaric acid tablets; formulation B: syloid tablets.
